# Megabenthic communities of the Ligurian deep continental shelf and shelf break (NW Mediterranean Sea)

**DOI:** 10.1371/journal.pone.0223949

**Published:** 2019-10-17

**Authors:** Francesco Enrichetti, Carlos Dominguez-Carrió, Margherita Toma, Giorgio Bavestrello, Federico Betti, Simonepietro Canese, Marzia Bo

**Affiliations:** 1 Dipartimento di Scienze della Terra, dell'Ambiente e della Vita, Università degli Studi di Genova, Genova, Italy; 2 Okeanos Research Centre, Universidade dos Açores, Departamento de Oceanografia e Pesca, Horta, Portugal; 3 IMAR Instituto do Mar, Universidade dos Açores, Departamento de Oceanografia e Pesca, Horta, Portugal; 4 Istituto Superiore per la Protezione e la Ricerca Ambientale, Roma, Italy; Australian Bureau of Agricultural and Resource Economics and Sciences, AUSTRALIA

## Abstract

The Ligurian Sea is one of the most studied Mediterranean basins. Since the beginning of the last century, many research expeditions have characterized its benthic and pelagic fauna through scuba diving and trawl surveys. However, a large knowledge gap exists about the composition of benthic communities extending into the so-called mesophotic or twilight depth range, currently under intense pressure from commercial and recreational fishing. A series of visual surveys, carried out by means of remotely operated vehicles between 2012 and 2018, were conducted along the Ligurian deep continental shelf and shelf break, between 30 and 210 m depth, in order to characterize the main benthic biocoenoses dwelling at this depth range and to determine the most relevant environmental factors that explain their spatial distribution. Deep circalittoral communities of the Ligurian Sea were represented by a mixture of species belonging to the deepest extension of shallow-water habitats and deep circalittoral ones. Twelve major biocoenoses were identified, each one characterized by specific preferences in depth range, substrate type and seabed slope. Those biocoenoses included gorgonian and hydrozoan forests, dense keratose sponge grounds, *Dendrophyllia cornigera* gardens, bryozoan beds and soft-bottom meadows of sabellid polychaetes and soft-corals. Other less common aggregations included six forests of black corals and two populations of *Paramuricea macrospina*. A georeferenced database has been created in order to provide information to managers and stakeholders about the location of the identified communities and high-diversity areas, aiming to facilitate sustainable long-term conservation of the Ligurian benthic ecosystem.

## Introduction

The Ligurian Sea, located in the northernmost part of the western Mediterranean, is a very well-studied area, with descriptions of its benthic fauna dating back to the 1800s [1,2,3]. During the last century, our knowledge about Ligurian benthic ecosystems has increased significantly, leading to the production of many detailed faunal catalogues, both for fishes and benthic invertebrates [4]. Several studies have focused on the diversity and distribution of megabenthic organisms in coastal areas [5,6,7,8], paying particular attention to seagrass meadows [9,10,11] and shallow coralligenous reefs [12,13,14]. As a result, shallow-water megabenthic communities are now widely described and spatially mapped [15]. The deep-sea megafauna (>200 m) has also been partially characterized, mainly through a long history of scientific observations of the trawling discards [16,17,18,19]. Indeed, the cold-water coral banks located along the Ligurian continental slope, and recorded in the fishing bycatch, were already tentatively mapped by Fusco in 1968 [20,21].

In contrast, less attention has been given to the deep Ligurian continental shelf (~40–120 m) and shelf break (~120–200 m), corresponding to the “circalittoral” plane [22], also known as the twilight zone or mesophotic zone [4,23]. The exploration of such environments in the Mediterranean Sea has increased in recent years thanks to the progress made in acoustic mapping techniques, the availability of high definition (HD) video platforms (such as remotely operated vehicles, ROVs) and deep scuba procedures [24,25,26,27,28]. These studies highlight the presence of rich megabenthic communities on the whole extension of the continental shelf, some of which fall within the definition of “animal forests” [29]. Gorgonians, soft corals, sea pens, black corals, hydrozoans, sponges and large bryozoans constitute the main structuring components of such Mediterranean communities [28]. Due to their complex three-dimensional shapes, which enhance spatial and functional heterogeneity [30,31], marine animal forests attract a rich associated fauna [25,29,32] and play a significant role in benthic-pelagic coupling, providing a strong link between the water column and the seafloor environment [33,34].

Recent studies have identified a wide array of human activities that put the survival of Mediterranean benthic communities at risk, namely demersal fishing, pollution, construction of littoral infrastructures and seafloor drilling, among others [28,35,36,37,38]. Fishing pressure is widely distributed in the western Mediterranean and currently represents the most critical threat for the long-term conservation of benthic habitats. Demersal gears in particular remove or damage benthic organisms, which due to their shape and size can get easily entangled in nets and longlines [39,40,41]. Furthermore, the contact with lost fishing gears can enhance wounds and the overgrowth of epibionts on coral structures, as well as the burial of sessile organisms due to sediment resuspension [28,37,42,43]. Considering that fishes and crustaceans of commercial interest are often associated with complex, well-structured benthic communities [44,45,46,47], the aggregation of commercial and recreational fishing in species-rich areas threatens the survival of these organisms [28,37].

In this context, an extensive exploration of the megabenthic communities found on the Ligurian mesophotic ecosystems was of primary interest to understand the distribution and characteristics of its natural resources. This study aims to fill the knowledge gap about the diversity of benthic communities found on the Ligurian deep continental shelf and shelf break, between 30 and 200 m depth, based on a community analysis and a large-scale geographical and bathymetrical distribution, together with an assessment of the environmental factors that influence such distribution.

## Materials and methods

### Ethics statement

This research has been commissioned and approved by the Ministero dell'Ambiente e della Tutela del Territorio e del Mare (MATTM) and Agenzia Regionale per la Protezione dell'Ambiente Ligure (ARPAL). The following is a summary of the non-invasive benthic imagery surveying and subsequent imagery annotation. Neither endangered or protected species were sampled within this study.

### Study area

**Fig 1:**
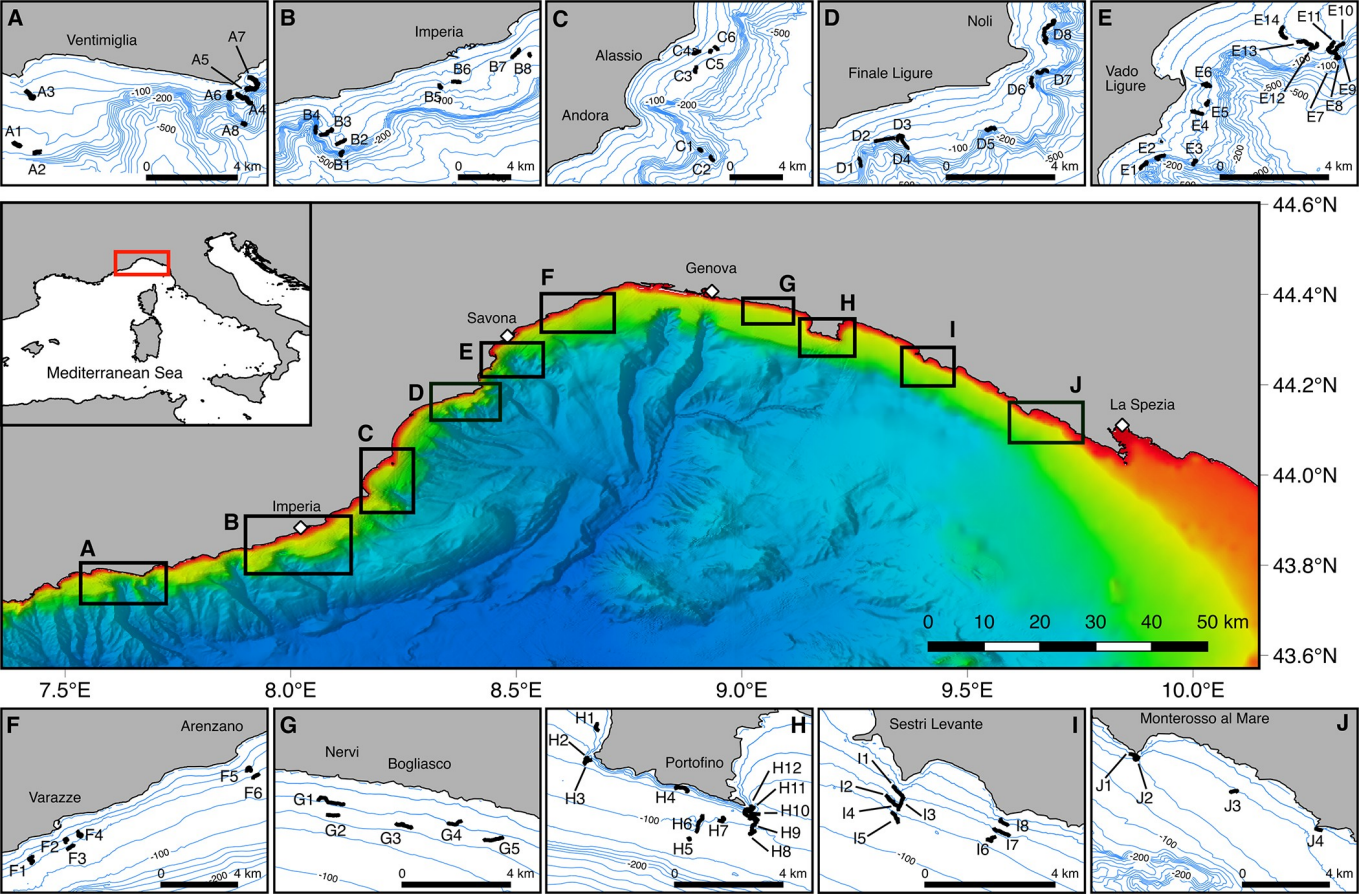
Study area. Map of the Ligurian Sea displaying the location of the 80 ROV dives carried out on the Ligurian continental shelf and shelf break. The 10 study areas selected for this study are shown as black boxes (A-J). Base bathymetry was downloaded from http://www.emodnet-bathymetry.eu.

The Ligurian Sea embraces the north-western sector of the Mediterranean Sea, extending from the Gulf of Lions to the northern Tyrrhenian Sea, and bordered on the south by Corsica Island. This study mainly focuses on the deep continental shelf of the Liguria coastline, extending for over 350 km, from Ventimiglia to La Spezia (Fig 1). The seabed topography of the Ligurian Sea consists of two sub-basins. The western basin is deeper, reaching depths of 2,600 m, and presents a steep and narrow shelf incised by 16 submarine canyons [4,48,49]. Upwelling currents occur within this sector, enhanced by the common occurrence of mesoscale anticyclonic eddies, and resulting in considerable input of nutrients in the euphotic layer [50]. In the eastern sector, the continental shelf is wider, with a less pronounced slope and with only three major canyon systems. The majority of the Ligurian canyons are shelf-incising, with no clear bathymetric connection to a major river system [51].

Due to the cyclonic circulation, the overall superficial water flow moves westward along the basin [4]. Intermediate Levantine waters and deep waters entering the basin from the Tyrrhenian Sea through the Tuscan Archipelago and along the western coasts of Corse follow a similar cyclonic path [4,52,53]. More than 76 rivers, with predominantly torrential streams, flow through the Alpine and Apennine chains within the investigated portion of the Ligurian basin. Magra River, flowing in the easternmost part of the study area, represents the largest Ligurian river and causes the seawater to be turbid and rich in nutrients in the eastern sector [54,55].

### Video survey

Due to the large extent of the Ligurian Riviera, video sampling was concentrated in ten major areas (from A to J moving eastward; Fig 1), where a total of 80 underwater video transects were carried out (see S1 Table for details of each dive). 70 transects were recorded during three surveys conducted on board of the R/V *Astrea* ISPRA (Rome) in 2012, 2015 and 2016. The ROV Pollux III was equipped with a digital camera (Nikon D80), a strobe (Nikon SB 400), a HD video camera (Sony HDR-HC7), underwater lights and a 3-jaw grabber. The ROV also had a depth sensor, a compass and the underwater acoustic positioning system (Ultra-Short Baseline, USBL) LinkQuest TrackLink 1500 MA to obtain accurate georeferenced positions every second when underwater. Parallel laser beams provided a scale for dimensional reference (8 cm in width). The remaining 10 transects were recorded in 2018 by COLMAR S.r.l. Technological Development Center (La Spezia), employing a BlueROV2, equipped with an HD video camera, underwater lights, parallel laser beams (13 cm in width), USBL Sonardyne Scout Plus, a depth sensor and a compass. In order to guarantee similar video quality for all video footage, both ROVs aimed to move at a constant speed of less than 0.3 m·sec^-1^ and a distance from the seabed lower than 1.5 m [56,57]. The average duration and length of the video transects was 45 minutes and around 750 m, with depths ranging between 28 and 216 m (details in S1 and S2 Tables). Voucher samples for those species for which identification through the video footage was difficult were collected by means of a grabber and subsequently dried or fixed in absolute ethanol for further taxonomic identification. Collected samples did not included endangered or protected species.

### Video analysis

Video images were evaluated using Apple’s Final Cut Pro X software (version 10.4) following the methodology described by Gori et al. [58]. Loops and pauses during the ROV transect were erased in order to obtain a linear video track. After smoothing the ROV trajectory provided by the raw GPS positioning, track length was measured with the open-source software Quantum GIS (version 2.18) [59]. Sequences too far away from the seafloor or with poor image quality were considered unsuitable for the analysis. The valid footage accounted for approximately 83% of the recorded material, corresponding to a length of about 51 km. All megabenthic organisms observed in the images within a visual field of 50 cm were annotated to the lowest possible taxon. Encrusting organisms were not considered in the analysis, due to the difficulty of identifying them from the video footage. The time elapsed since the beginning of the dive was assigned to each organism, and time was later converted to distance using the speed of the ROV derived from the acoustic position system. Substrate type was also identified along the transect using five different categories (describing the dominant granulometry as detectable from ROV images): (1) muds and fine sand, (2) sand and gravel, (3) cobbles and pebbles, (4) outcropping rock and (5) coralligenous rock. Categories (1), (2) and (3) may also include small and sparse biogenic detritus or coral rubble. Each transect was then divided into a string of adjacent 5 m^2^ sampling units (SU). The size of the SU was chosen following the results of Grinyó et al. [56] and Dominguez-Carrió [57], which evaluated areas of a similar depth range in the western Mediterranean. The number of organisms of each species was assigned to all SU, together with the percentage of each substrate category. Depth, slope and seafloor roughness values (the difference between the maximum and the minimum value of a given cell and its 8 surrounding cells) were calculated from the multibeam bathymetry specifically collected at each diving site using the location of the middle point of the SU.

### Data analyses

#### Megabenthic diversity

In order to characterize the diversity of the benthic habitats along the whole study area, differences among sites were investigated in terms of species richness, diversity and abundance. Species richness was calculated as number of conspicuous animal taxa per SU (excluding highly mobile taxa such as fishes and cephalopods), equally considering organisms identified as morphospecies under different taxonomic levels (species, genus or higher taxa). Megabenthic diversity was estimated using the exponential of the Shannon diversity index, which provides an indication of the effective number of species per sample and has the same linear metric as species richness [60]. Finally, local densities were calculated considering the number of organisms per SU.

#### Community analysis

Aiming to identify the major megabenthic communities of the Ligurian continental shelf and shelf break, those species with less than 10 individuals across the whole survey area were considered rare and removed from the multivariate analyses. Furthermore, to reduce the background noise produced by the large amount of SU with low abundances, only samples with an average density of more than 2 org·m^-2^ were considered for the statistical analyses. Associations of species were determined by means of the clustering algorithm Ward’s minimum variance method, constructed over square-root Bray-Curtis dissimilarity measures that were previously calculated from the square-root transformed density data. Samples belonging to the massive aggregation of the sabellid polychaete *Bispira viola* (Grube, 1863), with densities in most cases over two orders of magnitude higher than the remaining species, were considered independently and not included in the clustering analysis. The hierarchical dendrogram was built using the function *hclust* of the package *stats* in the R environment. The optimal number of groups in which to split the dataset was determined by selecting the highest average silhouette width from all cluster solutions using the *silhouette* function included in the *cluster* package [61]. Once groups were identified, distance-based permutational multivariate analysis of variance (PERMANOVA) was run to determine differences statistically significant between groups based on their species composition using the *adonis* function included in the *vegan* package [62]. The relative importance of all taxa in each community was determined using the Indicator Value (IndVal), which measures the fidelity (relative frequency) and specificity (relative abundance) of species within groups in order to identify those that can be considered characteristic of each association [63]. IndVal measures were computed using the function *indval* included in the *labdsv* package [64]. To better understand the influence of the selected environmental parameters (depth, slope, substrate type and seabed roughness) in determining differences in species composition, a distance-based redundancy analysis (dbRDA) was computed over the square-root of Bray-Curtis dissimilarity measures and the environmental matrix with standardized values using the function *capscale* included in the *vegan* package [62].

#### Geographic distribution

Based on the geographical position of each SU, the spatial distribution of all communities was mapped using the open-source software Quantum GIS. To provide a better representation of the importance of structuring species within the diversity of the megabenthic communities, the density and geographical distribution of the most relevant species observed in the video footage was also represented in distribution maps, providing a valuable and effective tool for future monitoring and conservation programs. In addition to the main species that emerged from the community analysis, other structuring organisms also considered relevant (importance within low-density populated environments and/or rarity) were further evaluated.

## Results

### General results

The exploration of the Ligurian continental shelf provided a total of 63 hours of video footage, corresponding to an explored area of 31,000 m^2^ of seabed (S1 Table). Excluding the SU characterized by bad visibility or excessive distance to the seabed, 3,747 SU were suitable for statistical analyses, corresponding to a total usable area of about 19,000 m^2^. Soft bottoms, mainly represented by mud and fine sands, were the most widespread, occupying 63% of the total explored area. The remaining 37% corresponded to hard bottoms, that being cobbles and pebbles (1.4%), coralligenous rocks (9%) and outcropping rocks (27%). A comprehensive description of the environmental features that characterized each ROV dive is provided in S2 Table.

**Fig 2:**
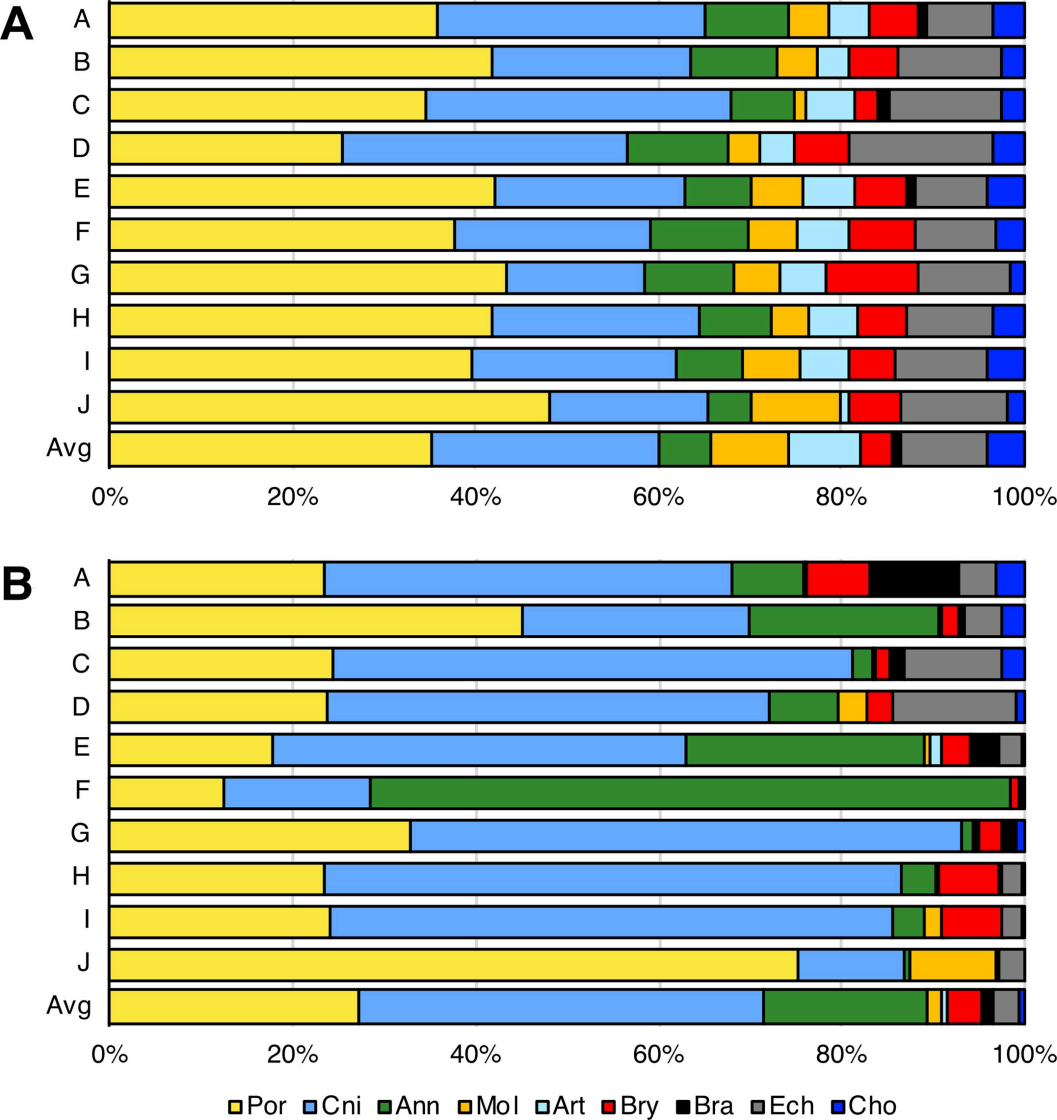
Biodiversity of megafauna species along the Ligurian coast. Percentage of the number of species (A) and abundance (B) of megafauna per phylum in each of the 10 study areas (A: Ventimiglia; B: Imperia; C: Alassio; D: Finale Ligure; E: Savona; F: West Genova; G: East Genova; H: Portofino; I: Sestri Levante; J: Cinque Terre). Por: Porifera; Cni: Cnidaria; Ann: Annelida; Mol: Mollusca; Art: Artropoda; Bry: Bryozoa; Bra: Brachiopoda; Ech: Echinodermata; Cho: Chordata.

A total of 219,029 megabenthic organisms, belonging to 224 different taxa, were identified in the video images (S3 Table). From this list, 72% of the taxa could be identified to species (51%) or genus level (21%), whereas the remaining 62 morphospecies had to be classified in higher taxonomic levels: family (2%), order (3%), class (2%) or phylum (18%). Sponges and corals represented a major component of Ligurian benthic communities, contributing almost 60% of the total number of taxa identified (Fig 2A). To a lesser extent, echinoderms (9.5%), molluscs (8.6%), crustaceans (7.7%), annelids (5.9%) and ascidians (4.1%) also contributed to the overall megafauna diversity of the study area. Cnidarians were the most abundant group in terms of number of organisms (44%), followed by sponges (27%) and annelids (18%) (Fig 2B). The remaining phyla contributed to approximately 10% of the total number of organisms. The yellow sponges *Axinella* spp., often in association with the zoanthid *Parazoanthus axinellae* (Schmidt, 1862) represented both the most abundant and the most widely distributed organisms, with 34,314 records and appearing in 89% of the dives. The scleractinian *Leptopsammia pruvoti* Lacaze-Duthiers, 1897 and the annelid *Bispira viola* (Grube, 1863) were also very abundant along the Ligurian coast. In terms of spatial occurrence, the gorgonians *Eunicella verrucosa* (Pallas, 1766) and *Paramuricea clavata* (Risso, 1826) were the most widely distributed species besides the complex *Axinella* spp./*Parazoanthus axinellae*.

#### Regional differences

The amount of investigated area ranged from 500 m^2^ in Area J (Cinque Terre) to about 3000 m^2^ in Area E and H (Savona and Portofino, respectively) (Table 1). The four areas located in the easternmost sector of the Ligurian Sea were generally more homogeneous and shallower than the other areas. An accurate description of the biological features for each ROV dive is provided in S4 Table. Overall, the average number of megabenthic organisms per SU was 38 (± 138), with an average number of species of 3.97 (± 4.14). The number of species identified in each dive was very inconsistent throughout the whole Ligurian continental shelf, varying between 8 (Pora Canyon) and 80 (Bordighera). Areas B (Imperia) and E (Savona), both located in the westernmost sector, had on average the highest number of species per SU and also the highest species richness and diversity based on the exponential of the Shannon index (Table 1). The lowest values of species richness and diversity were observed in Area G (East Genova), which is located in the eastern sector. Area E (Savona) had, on average, the highest number of organisms per SU (92 ± 240), with 2, 758 organisms identified in one single dive (Vado shoals).

**Table 1:** Overall differences between the 10 study areas in terms of species composition and diversity of the invertebrate megafauna.

Area	N° dives	Area explored (m^2^)	Depth range (m)	Av. n° spp. per SU	Av. expH per SU	Av. n° org. per SU	Number of species								
							Por	Cni	Ann	Mol	Art	Bry	Bra	Ech	Cho
A	8	1845	28–200	4.0 ± 4.0	2.9 ± 2.2	19.8 ± 32.0	40	33	10	5	5	6	1	8	4
B	8	1340	33–154	6.2 ± 4.4	3.9 ± 2.6	46.0 ± 82.7	48	25	11	5	4	6	0	13	3
C	6	1435	28–216	2.8 ± 3.3	2.2 ± 1.9	17.5 ± 34.3	26	25	5	1	4	2	1	9	2
D	8	2345	71–141	2.9 ± 2.7	2.3 ± 1.7	12.0 ± 16.8	21	26	9	3	3	5	0	13	3
E	14	2950	36–151	6.3 ± 5.2	3.5 ± 2.4	91.6 ± 240	52	26	9	7	7	7	1	10	5
F	6	1715	34–63	3.5 ± 3.9	2.6 ± 2.0	64.3 ± 281	35	20	10	5	5	7	0	8	3
G	5	1605	30–57	2.3 ± 3.1	2.0 ± 1.4	18.8 ± 42.5	26	9	6	3	3	6	0	6	1
H	13	3115	29–106	3.8 ± 4.0	2.5 ± 1.8	34.2 ± 74.0	48	26	9	5	6	6	0	11	4
I	8	1885	33–85	3.0 ± 3.3	2.5 ± 2.0	15.0 ± 31.1	50	28	9	8	7	6	0	13	5
J	4	500	28–55	3.6 ± 3.8	2.7 ± 1.8	33.8 ± 61.0	50	18	5	10	1	6	0	12	2

Specificities of each area explored based on the taxonomic composition of its megafauna species. SU: sampling unit; Por: Porifera; Cni: Cnidaria; Ann: Annelida; Mol: Mollusca; Art: Arthropoda; Bry: Bryozoa; Bra: Brachiopoda; Ech: Echinodermata; Cho: Chordata.

In general terms, sponges dominated species composition, while cnidarians were the most abundant in the 10 investigated areas (Fig 2). Large differences in species diversity and abundance were observed between Area C (Alassio) and D (Finale Ligure) (where cnidarians contributed equally or more than sponges to the total biodiversity of the region, Fig 2A), Area B (Imperia) and J (Cinque Terre) (where sponges were the most abundant organisms), and Area F (West Genova) (where annelids represented up to 70% of the total number of organisms, Fig 2B).

### Benthic communities

**Fig 3:**
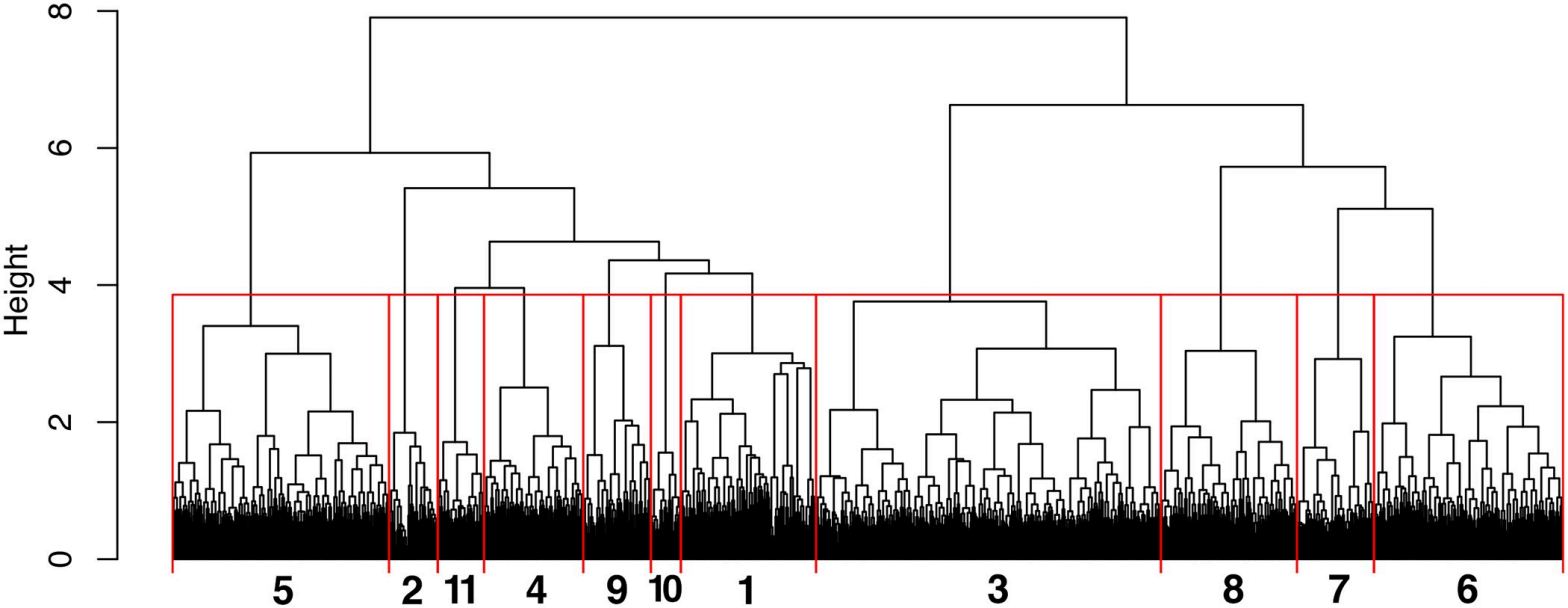
Cluster analysis. Dendrogram using Ward’s clustering method constructed over the square-root of Bray-Curtis dissimilarity measures of square-root transformed density data for all megabenthic species.

**Fig 4:**
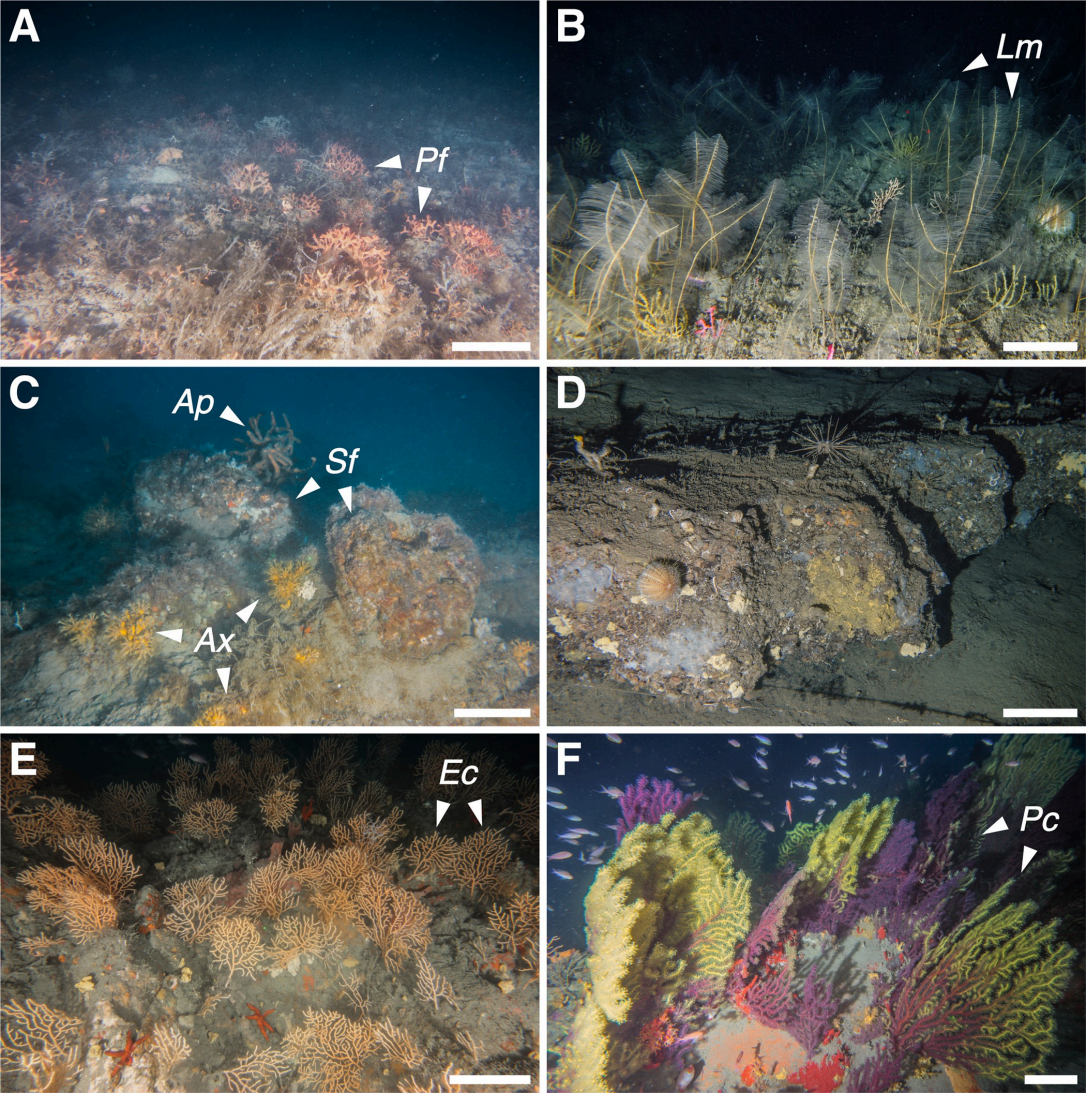
Megabenthic communities (part one). (A) 1—Bryozoan beds dominated by *P*. *fascialis* (*Pf*); (B) 2 –*L*. *myriophyllum* (*Lm*) forests; (C) 3—Sponge grounds with *S*. *foetidus* (*Sf*), *Axinella polypoides* (*Ap*) and *Axinella* spp. (*Ax*) covered with *P*. *axinellae*; (D) 4—Deep-rocky bottoms with serpulids and echinoderms; (E) 5 –*E*. *cavolini* (*Ec*) forests; (F) 6 –*P*. *clavata* (*Pc*) forests. Scale bar 20 cm.

**Fig 5:**
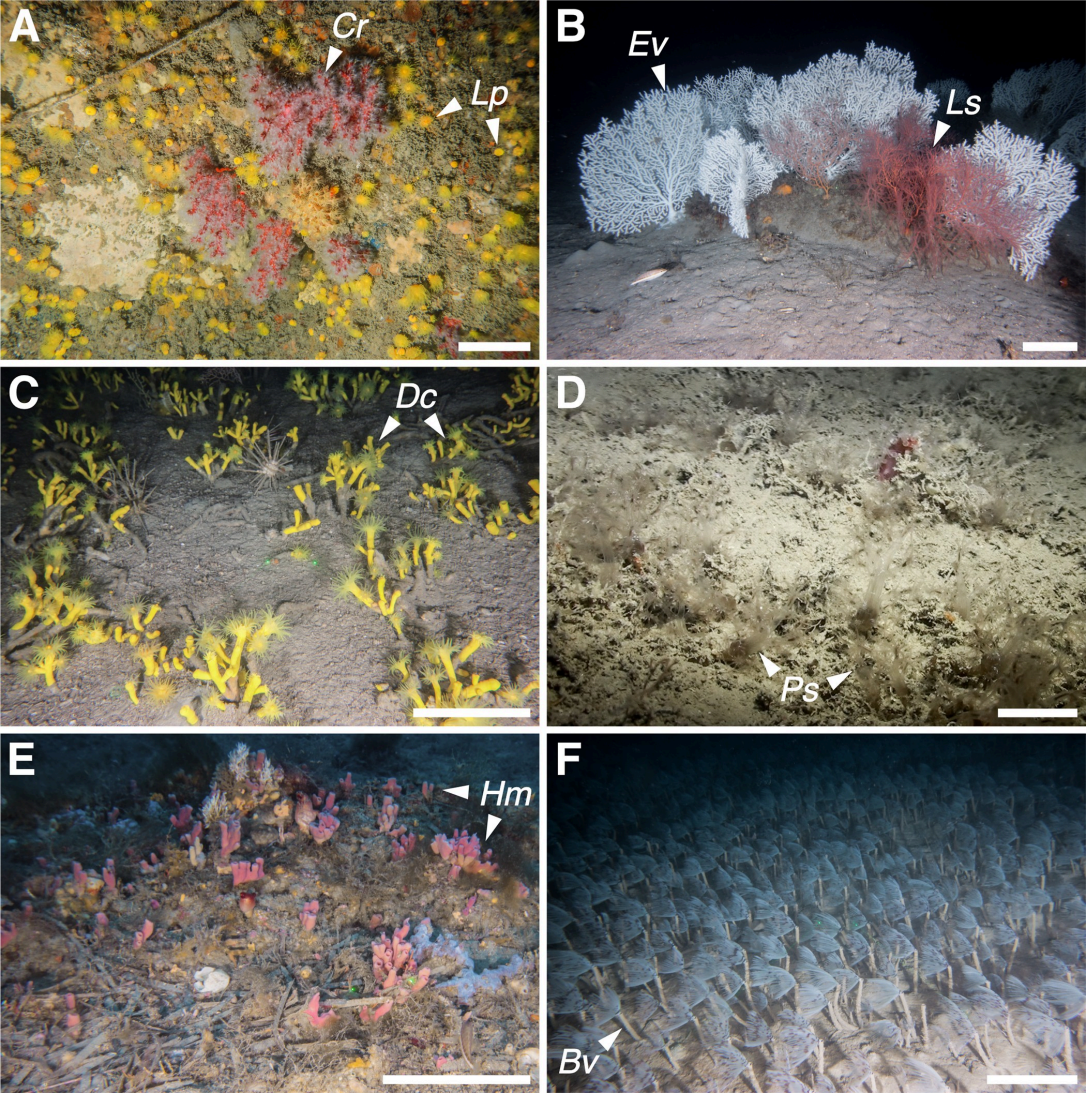
Megabenthic communities (part two). (A) 7—Coralligenous overhangs hosting by *L*. *pruvoti* (*Lp*) and *C*. *rubrum* (*Cr*); (B) 8 –*E*. *verrucosa* (*Ev*) forests with colonies of *L*. *sarmentosa* (*Ls*); (C) 9 –*D*. *cornigera* (*Dc*) gardens; (D) 10 –*P*. *spinulosum* (*Ps*) meadows; (E) 11—*Haliclona* cf. *mediterranea* (*Hm*) fields; (F) 12 –*B*. *viola* (*Bv*) beds. Scale bar 20 cm, except for A and D of 5 cm.

22% of the suitable SU for statistical analyses (828) did not contain a single megabenthic organism and 45% (1692) contained more than 10. Considering the dense aggregation of the sabellid *B*. *viola* as a separate community, the clustering of the remaining SU in a hierarchical dendrogram provided a total of 11 major megabenthic associations (Table 2, Fig 3), significantly different from each other based on species composition (p-perm<0.01). A comprehensive list of the most important species characterizing each community based on the IndVal index is provided in Table 3. A species/taxa name, generally based on the characteristic species, was used to designate each community in order to facilitate reading (Figs 4 and 5). A description of each community identified follows.

**Table 2:** Main features of the 12 communities identified in the continental shelf and shelf break of the Ligurian Sea.

ID	Characteristic species	% SU	Av. n° spp. per SU ± sd	Av. n° org. per SU ± sd	Av. expH per SU ± sd	Substrate type (%)					Depth (m)	
						Mud/ Sand	Sand/ Gravel	Cobb./ Pebbl.	Rock	Cor. Rock	Min	Max
1	*P*. *fascialis*	9.59	6.2 ± 3.8	9.7 ± 19.7	4.0 ± 2.7	55	8	0.5	25	7	30	106
2	*L*. *myriophyllum*	3.46	3.6 ± 2.2	4.7 ± 3.1	2.3 ± 1.2	87			11	1	49	102
3	*Axinella* spp.	24.45	8.0 ± 3.4	12.1 ± 8.6	4.9 ± 1.9	25	3	0.5	40	30	28	83
4	Serpulidae	9.85	6.7 ± 2.7	8.9 ± 18.8	4.2 ± 1.8	16	0.2	1	79	4	34	208
5	*E*. *cavolini*	15.35	9.2 ± 3.4	15.0 ± 10.6	4.8 ± 1.7	11	0.3	1	83	5	31	129
6	*P*. *clavata*	13.41	7.4 ± 3.0	9.7 ± 6.3	4.6 ± 2.0	17	2		50	31	29	89
7	*L*. *pruvoti*	5.46	12.1 ± 5.9	63.8 ± 44.7	3.9 ± 1.8	8	5	2	50	36	30	85
8	*E*. *verrucosa*	9.65	6.7 ± 3.3	5.2 ± 2.6	4.4 ± 2.3	50		3	45	2	30	92
9	*D*. *cornigera*	1.88	3.0 ± 1.5	6.5 ± 3.4	2.0 ± 1.0	74			26		84	209
10	*P*. *spinulosum*	2.12	3.9 ± 2.1	19.1 ± 14.2	1.6 ± 0.8	96			4		68	106
11	*H*. cf. *mediterranea*	3.28	6.6 ± 2.7	10.2 ± 7.6	3.4 ± 1.6	14	72		8	7	46	88
12	*B*. *viola*	1.40	4.5 ± 3.4	263.9 ± 183	1.3 ± 0.7	79			19		56	69

Characteristic species correspond to those scoring higher in the IndVal analysis, with the exception of community 3 where the most abundant species was selected as characteristic (see main text for further information). SU: sampling unit.

**Table 3:** Summary of the main megabenthic species identified in this study.

Community	Species	IndVal	Avg. density (org·m^-2^ ± sd)	Max. density (org·m^-2^ ± sd)
**1**	*Pentapora fascialis*	0.16	0.89 ± 2.02	14
	*Neopycnodonte cochlear*	0.12	1.61 ± 6.73	49
	*Filograna/Salmacina* complex	0.09	0.62 ± 2.04	16.4
	Porifera sp. 57	0.09	0.32 ± 1.48	12.8
	*Myriapora truncata*	0.07	0.33 ± 1.24	7.8
	*Reteporella* spp.	0.05	0.14 ± 0.3	2
	*Alcyonium acaule*	0.05	0.09 ± 0.55	6.4
**2**	*Lytocarpia myriophyllum*	0.84	3.04 ± 1.55	7.2
	*Filograna/Salmacina* complex	0.06	0.33 ± 0.84	5
	*Smittina cervicornis/Adeonella calveti*	0.05	0.16 ± 0.26	1
**3**	*Parazoanthus axinellae*	0.36	3.75 ± 3.45	19.2
	*Sarcotragus foetidus*	0.21	0.39 ± 0.56	2.8
	*Dysidea* sp.	0.21	0.25 ± 0.65	7.6
	*Axinella* spp.	0.19	4.3 ± 3.73	25.4
	*Axinella polypoides*	0.11	0.09 ± 0.03	3.8
	*Eunicella verrucosa*	0.10	0.73 ± 1.22	8.6
	*Halocynthia papillosa*	0.06	0.21 ± 0.5	4.6
	*Chondrosia reniformis*	0.05	0.13 ± 0.56	8.2
	*Bonellia viridis*	0.05	0.08 ± 0.2	2
**4**	Serpulidae	0.27	1.23 ± 3.69	26.4
	*Holothuria* spp.	0.15	0.36 ± 0.48	2.8
	*Cidaris cidaris/Stylocidaris affinis*	0.14	0.47 ± 0.66	4
	Porifera sp. 49	0.11	0.21 ± 0.44	3
	*Echinus melo/Gracilechinus acutus*	0.08	0.05 ± 0.12	0.6
	*Apomatus/Protula* complex	0.07	0.06 ± 0.15	0.8
	*Axinella* spp.	0.07	1.33 ± 1.41	6.2
	*Megerlia truncata*	0.06	0.85 ± 4.39	40
**5**	*Eunicella cavolini*	0.60	4.95 ± 4.89	22.4
	*Alcyonium coralloides*	0.27	0.53 ± 1.04	8.4
	*Axinella* spp.	0.12	3.11 ± 3.77	20.4
	*Hexadella racovitzai*	0.08	0.13 ± 0.29	2.2
	*Astrospartus mediterraneus*	0.07	0.07 ± 0.21	1.6
	*Holothuria* sp.	0.06	0.21 ± 0.37	2.4
	Porifera sp. 49	0.06	0.19 ± 0.52	3.6
	*Antedon mediterranea*	0.05	0.22 ± 0.88	8.2
**6**	*Paramuricea clavata*	0.35	3.51 ± 3.33	18.8
	*Aplysina cavernicola*	0.09	1.05 ± 2.43	16.8
	*Axinella* spp.	0.08	1.42 ± 1.55	6.8
	*Eunicella verrucosa*	0.07	0.61 ± 1.36	15.04
	*Filograna/Salmacina* complex	0.06	0.34 ± 0.78	5.4
	*Turbicellepora* sp.	0.05	0.17 ± 0.41	2.6
**7**	*Leptopsammia pruvoti*	0.97	38.67 ± 33.62	143
	*Corallium rubrum*	0.49	3.98 ± 8.64	8.6
	*Agelas oroides*	0.26	0.39 ± 0.72	2.8
	*Petrosia ficiformis*	0.25	0.77 ± 1.89	12.6
	*Paramuricea clavata*	0.24	4.05 ± 4.47	17
	*Axinella* spp.	0.23	6.68 ± 4.35	24.2
	*Aplysina cavernicola*	0.22	1.51 ± 2.29	10.4
	Porifera sp. 4	0.20	0.47 ± 0.89	4.6
	*Smittina cervicornis/Adeonella calveti*	0.16	1.1 ± 2.11	11
	*Parazoanthus axinellae*	0.14	2.16 ± 3	11.8
**8**	*Eunicella verrucosa*	0.41	2.3 ± 1.51	7.2
	*Crella* sp.	0.14	0.09 ± 0.2	1
	*Leptogorgia sarmentosa*	0.11	0.15 ± 0.47	3.8
	*Eunicella singularis*	0.10	0.17 ± 0.54	3.4
	*Turbicellepora* sp.	0.06	0.15 ± 0.33	2.4
	*Frondipora verrucosa*	0.05	0.04 ± 0.14	1.4
	*Sarcotragus foetidus*	0.05	0.14 ± 0.35	3.2
	*Reteporella* spp.	0.05	0.12 ± 0.22	1.2
	*Smittina cervicornis/Adeonella calveti*	0.05	0.22 ± 0.44	2.6
**9**	*Dendrophyllia cornigera*	< 0.05	5.01 ± 3.69	14.8
	*Cidaris cidaris/Stylocidaris affinis*	< 0.05	0.57 ± 0.76	2.8
	*Pachastrella monilifera*	< 0.05	0.12 ± 0.39	1.8
	*Gryphus vitreus*	< 0.05	0.19 ± 1.04	5.8
	*Myxicola* sp.	< 0.05	0.05 ± 0.11	0.4
	Scleractinia sp. 1	< 0.05	0.13 ± 0.48	2.4
	Scleractinia sp. 4	< 0.05	0.01 ± 0.05	0.2
**10**	*Paralcyonium spinulosum*	1.00	16.91 ± 14.65	76.6
	*Alcyonium palmatum*	0.06	0.09 ± 0.22	1.2
	*Acromegalomma* sp.	0.06	0.02 ± 0.06	0.2
**11**	*Haliclona* cf. *mediterranea*	0.89	5.47 ± 3.94	15.4
	*Amphiura* sp.	0.18	0.29 ± 0.62	3
	*Axinella* spp.	0.12	2.1 ± 2.72	13.6
	Porifera sp. 20	0.11	0.05 ± 0.15	0.6
	*Alcyonium palmatum*	0.11	0.11 ± 0.23	1
	Sabellidae	0.11	0.07 ± 0.14	0.6
	*Echinaster sepositus/Hacelia attenuata*	0.10	0.13 ± 0.2	1
	*Halocynthia papillosa*	0.07	0.19 ± 0.43	2.2
**12**	*Bispira viola*	N/A	261.32 ± 185.21	551.6

Main species characterizing each of the 12 communities identified in this study based on their IndVal value.

**Fig 6:**
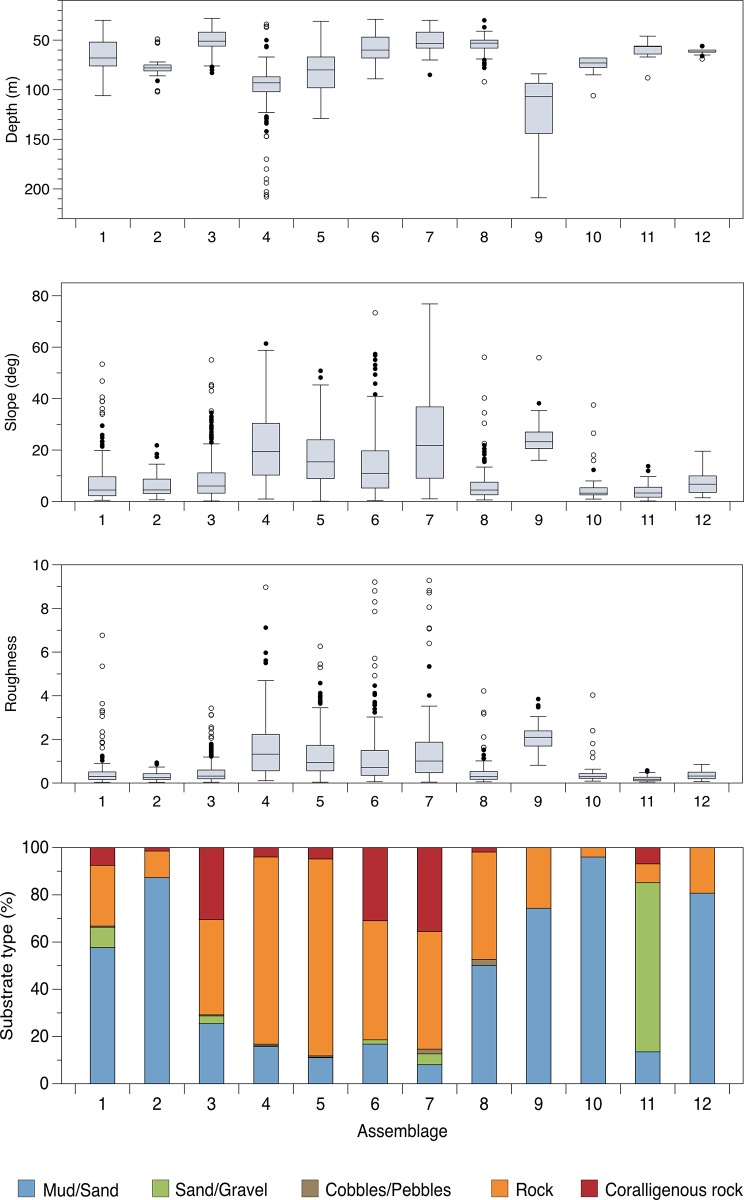
Environmental parameters. Range of depth, slope, roughness and substrate type for each community identified in the cluster analysis based on the information gathered for all sampling units used in the multivariate analyses.

**Fig 7:**
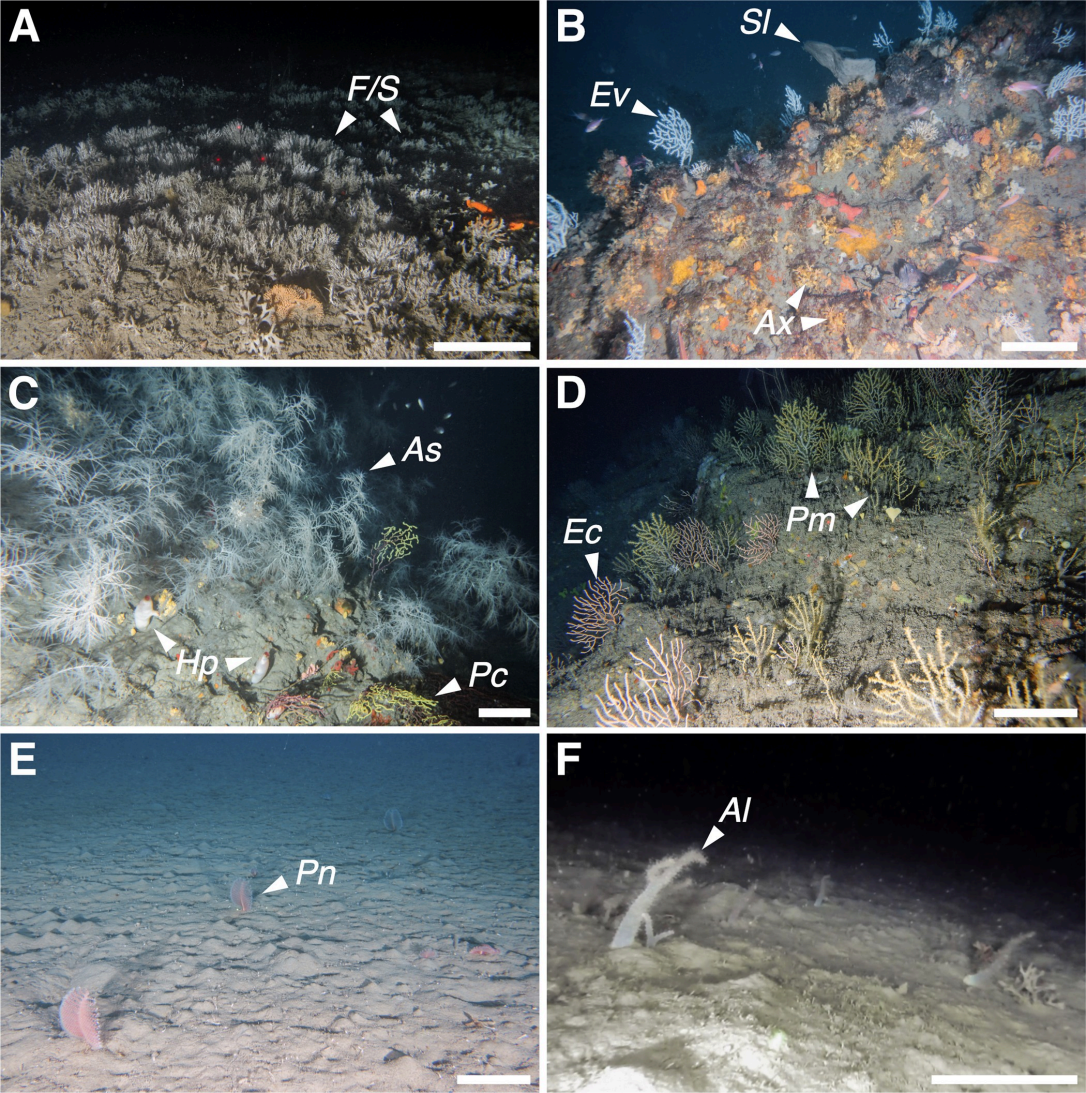
Additional megabenthic communities. (A) *Filograna/Salmacina* complex (*F/S*) field from Community 1. (B) A dense aggregation of *Axinella* individuals (*Ax*) covered with *P*. *axinellae* from Community 3. Some gorgonians (*E*. *verrucosa*, *Ev*) and a specimen of the keratose sponge *S*. *lamella* (*Sl*) are also present. C) *A*. *subpinnata* (*As*) forest, with some small specimens of *P*. *clavata* (*Pc*) and *H*. *papillosa* (*Hp*). D) Aggregation of *P*. *macrospina* (*Pm*), mixed with *E*. *cavolini* (*Ec*). E) *Pennatula* spp. (Pn) field. F) *A*. *palmatum* (*Al*) field. Scale bar: 20 cm.

Community 1: Bryozoan beds (Fig 4A). This community mainly occurred on horizontal soft bottoms located between 50 and 80 m depth (Fig 6). Its characteristic species was the erect bryozoan *Pentapora fascialis* (Pallas, 1766), with maximum densities of 14 col·m^-2^ (Table 3). The bivalve *Neopycnodonte cochlear* (Poli, 1795) also reached its highest densities within this community. It was found growing on wrecks and large metallic objects randomly distributed on the seabed, which in some cases were completely covered by those bivalves. Serpulids of the species complex *Filograna/Salmacina* were also predominant in certain spots, forming large fields (e.g. site of Savona, Fig 7A).

Community 2: Hydrozoan forests (Fig 4B). Large aggregations of the hydroid *Lytocarpia myriophyllum* (Linnaeus, 1758) (up to 7 col·m^-2^) occurred on horizontal grounds covered by muds and fine sands, mainly between 70 and 80 m depth (Fig 6). Some bryozoans and serpulids were often observed as epibionts of the large hydroid colonies.

Community 3: Sponge grounds (Fig 4C). Encountered on various substrate types, with highest abundances on flat hard bottoms between 40 and 60 m depth (Fig 6). This community corresponds to a multi-specific sponge aggregation, dominated by the large massive keratose Porifera *Sarcotragus foetidus* Schmidt, 1862 (densities up to 3.2 ind·m^-2^) together with *Dysidea* sp. (max. densities of 7.6 ind·m^-2^). The zoanthid *Parazoanthus axinellae* displayed the highest IndVal value in this community, although it was observed as an epibiont of the small yellow sponges *Axinella* spp. (Tables 2 and 3, Fig 7B). These sponges represented the most common organisms along the Ligurian continental shelf, reaching densities of 25 ind·m^-2^. Several other keratose sponges characterise this community, but their identification from the video footage was not always possible: six different morphotypes were recognised, with morphotypes 1, 2 and 4 being the most frequent. Rare and scattered large specimens of *Spongia (Spongia) lamella* (Schulze, 1879) were also observed, as well as the gorgonian *E*. *verrucosa*, the annelid *Bonellia viridis* Rolando, 1822 and the ascidiacean *Halocynthia papillosa* (Linnaeus, 1767).

Community 4: Deep-rocky bottom communities (Fig 4D). Steep rocks, mainly bare, usually colonized by unidentified serpulids worms and hosting several species of holothurians and sea urchins were observed along the whole study area. Their distribution covered a wide bathymetrical range, although mainly concentrated between 85 and 105 m depth (Fig 6). A large pseudo-encrusting unidentified sponge (Porifera sp. 49) was observed within this community, as well as the ubiquitous sponges *Axinella* spp. Dense patches of the brachiopod *Megerlia truncata* (Linnaeus, 1767) (up to 40 ind·m^-2^) were spotted in seven SU belonging to this community.

Community 5: *Eunicella cavolini* (Koch, 1887) forests (Fig 4E). This community occurred on sloping rocky bottoms between 65 and 100 m depth (Fig 6). Among the three gorgonian communities identified in the present study, *E*. *cavolini* forests (up to 22 col·m^-2^) represented the richest in terms of number of species and *expH* diversity (Table 2). The soft coral *Alcyonium coralloides* (Pallas, 1766) was often associated with gorgonian branches, whereas the sponges *Axinella* spp. and some holothurians were found inhabiting the understory. Large ophiuroids of the species *Astrospartus mediterraneus* (Risso, 1826) were also observed feeding at the top of the gorgonian colonies.

Community 6: *P*. *clavata* forests (Fig 4F). Aggregations characterized by the gorgonian *P*. *clavata* (densities up to 19 col·m^-2^) occurred on sloping outcroppings and coralligenous rocks generally between 45 and 65 m depth (Fig 6). These forests were also characterized by the presence of dense aggregations of the sponges *Aplysina cavernicola* (Vacelet, 1959) and *Axinella* spp., whereas in some cases other gorgonians such as *E*. *verrucosa* contributed to forming multi-specific gorgonian aggregations. Serpulids and bryozoans occupied the understory or were found as epibionts on the gorgonian branches.

Community 7: Coralligenous overhangings (Fig 5A). Sloping and vertical cliffs between 30 and 80 m depth hosted peculiar communities dominated by dense patches of the yellow scleractinian *L*. *pruvoti* (very high densities of up to 143 ind·m^-2^) and the precious red coral *Corallium rubrum* (Linnaeus, 1758) (up to 9 col·m^-2^). This community showed the highest average number of species per SU among all communities (Table 2). Several sponges were herein identified, namely *Agelas oroides* (Schmidt, 1864), *Petrosia (Petrosia) ficiformis* (Poiret, 1789), *Axinella* spp., *A*. *cavernicola*, together with colonies of the gorgonian *P*. *clavata*.

Community 8: *E*. *verrucosa* forests (Fig 5B). This community was characterized by monospecific or mixed aggregations, occurring on horizontal sub-outcropping rocks in high silted areas, where *E*. *verrucosa* reached densities up to 7 col·m^-2^. Depth ranged from 30 to 90 m, but the majority concentrated in a narrow bathymetrical range around 50 m (Fig 6). The sponges *Crella* sp. and *S*. *foetidus*, together with several species of bryozoans, were often observed. In some cases, *Leptogorgia sarmentosa* (Esper, 1789) occurred in multi-specific patches, becoming predominant in some localities (4 col·m^-2^).

Community 9: *Dendrophyllia cornigera* (Lamarck, 1816) gardens (Fig 5C). Aggregations of *D*. *cornigera* (up to 15 col·m^-2^) constituted the deepest community observed in this study, being identified down to 210 m depth, although its main distribution was between 90 and 145 m. The largest aggregation of this scleractinian coral occurred on muddy bottoms near rocky outcrops, but sparse colonies were also observed over rubble as well as outcropping rocks. Cidarids, the sponge *Pachastrella monilifera* Schmidt, 1868, the brachiopod *Gryphus vitreus* (Born, 1778), the sabellid *Myxicola* sp. and some solitary scleractinians were observed associated with this community. All species were characterized by low IndVal values, indicating a low specificity due to their presence in many other communities.

Community 10: *Paralcyonium spinulosum* (Delle Chiaje, 1822) meadows (Fig 5D). Few dense fields were found between 70 and 80 m depth on horizontal bottoms (Fig 6) dominated by mud, biogenic small detritus, as well as a biological cover of indeterminate algae, hydrozoans and polychaetes. Densities reached up to 77 col·m^2^. The soft coral *Alcyonium palmatum* Pallas, 1766 and some sabellids were occasionally associated with this community.

Community 11: *Haliclona* cf. *mediterranea* grounds (Fig 5E). This rare community was observed on horizontal bottoms dominated by coarse sands, gravels, outcropping rocks and biogenic detritus, mainly ranging between 55 and 65 m depth (Fig 6), where this sponge reached mean densities of 5 ind·m^-2^. Other species that characterise this community included the ophiuroid *Amphiura* sp., the sponges *Axinella* spp. and the soft coral *A*. *palmatum*.

Community 12: Sabellid beds (Fig 5F). This community occurred on horizontal muddy bottoms, often near *E*. *verrucosa* forests, in a narrow bathymetrical range, around 60–70 m depth (Fig 6). Here, the sabellid *B*. *viola* formed extraordinarily high-density aggregations (up to 550 ind·m^2^), displaying the highest average number of individuals per SU of all communities.

### Geographical distribution of megabenthic communities

**Fig 8:**
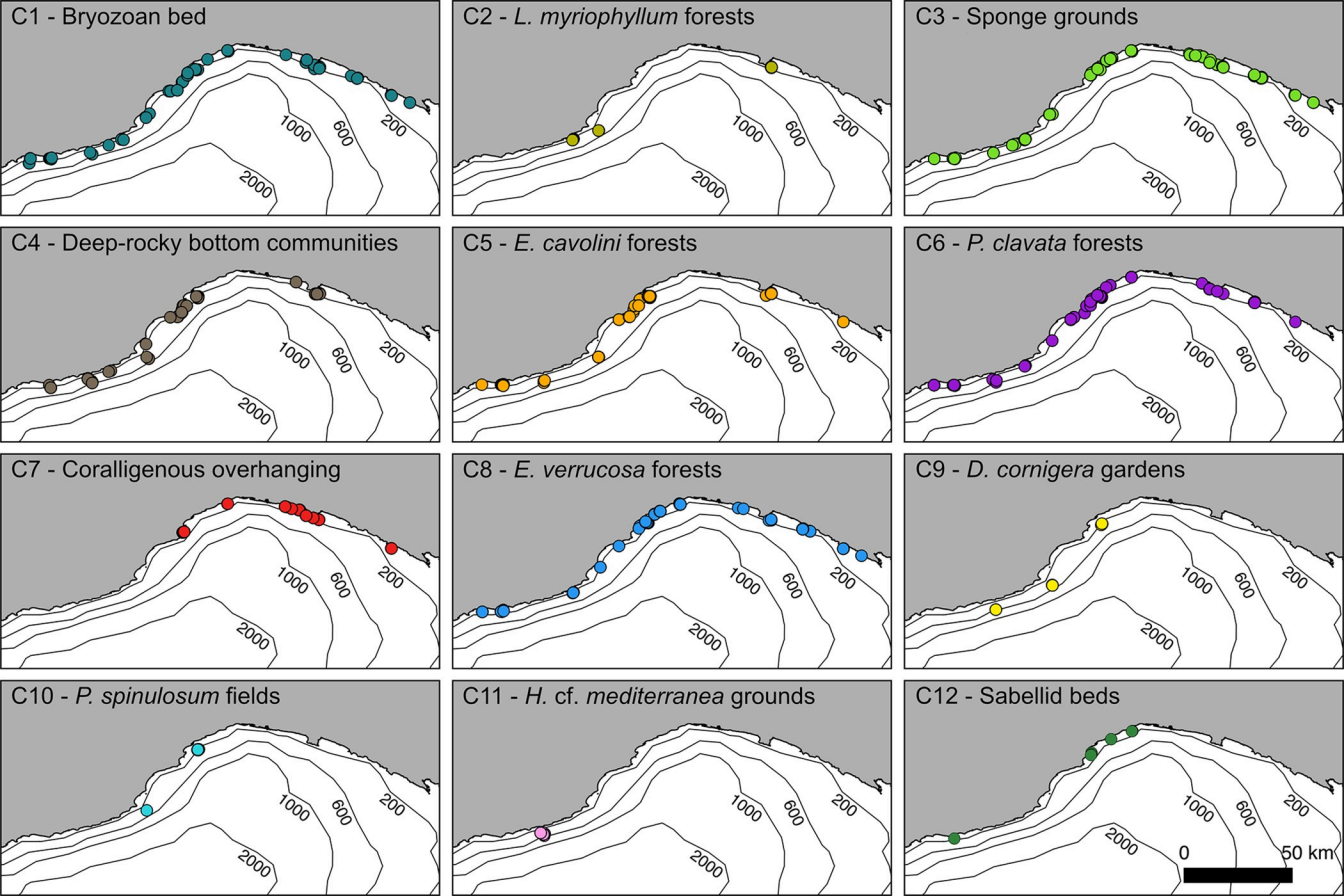
Distribution maps. Spatial distribution of the 12 megabenthic communities identified in the cluster analysis along the whole Ligurian coast.

The geographical distribution of the 12 communities identified in this study is shown in Fig 8. Six communities had a widespread distribution along the whole study area, including bryozoan beds, sponge grounds, deep-rocky bottom communities and gorgonian forests dominated by *E*. *cavolini*, *P*. *clavata* and *E*. *verrucosa*. Sponge grounds dominated by *S*. *foetidus* mainly occurred in the western Liguria Sea, whereas the sites of Mele Cape, Mantice Shoal and Punta del Faro hosted the densest forests of *E*. *cavolini* (up to 22 col·m^-2^). The best developed forests of *P*. *clavata* (up to 19 col·m^-2^) occurred in the sites of Maledetti Shoal, Savona shoals, Isuela and Punta Mesco. *E*. *verrucosa* was the most widespread gorgonian within the study area, reaching highest densities in the sites of Diano Marina, Finale Ligure and Arenzano (7 col·m^-2^).

The remaining communities showed a more scattered distribution pattern, being found in more specific locations. Large hydrozoan forests of *L*. *myriophyllum* occurred in the sites of Diano Marina, Mele Cape, Mantice Shoal and Punta del Faro. Deep vertical walls, hosting important populations of *L*. *pruvoti* and *C*. *rubrum* occurred at the Maledetti Shoal and Portofino Promontory. Low-density populations of red coral were found in other sites of the western Ligurian Sea, such as Bordighera, Santo Stefano and Finale Ligure. Community 9 was present in three sites, all of them located in the western Ligurian Sea, with the Mantice Shoal containing the largest aggregation of *D*. *cornigera*. *P*. *spinulosum* fields occurred only near the deep shoals of Mele Cape and Mantice, and the pink sponge *H*. cf. *mediterranea* dominated the sea bottoms of Santo Stefano. Finally, sabellid fields were observed in the sites of Bordighera, Vado Ligure, Varazze and Arenzano.

### Relationship with environmental parameters

**Fig 9:**
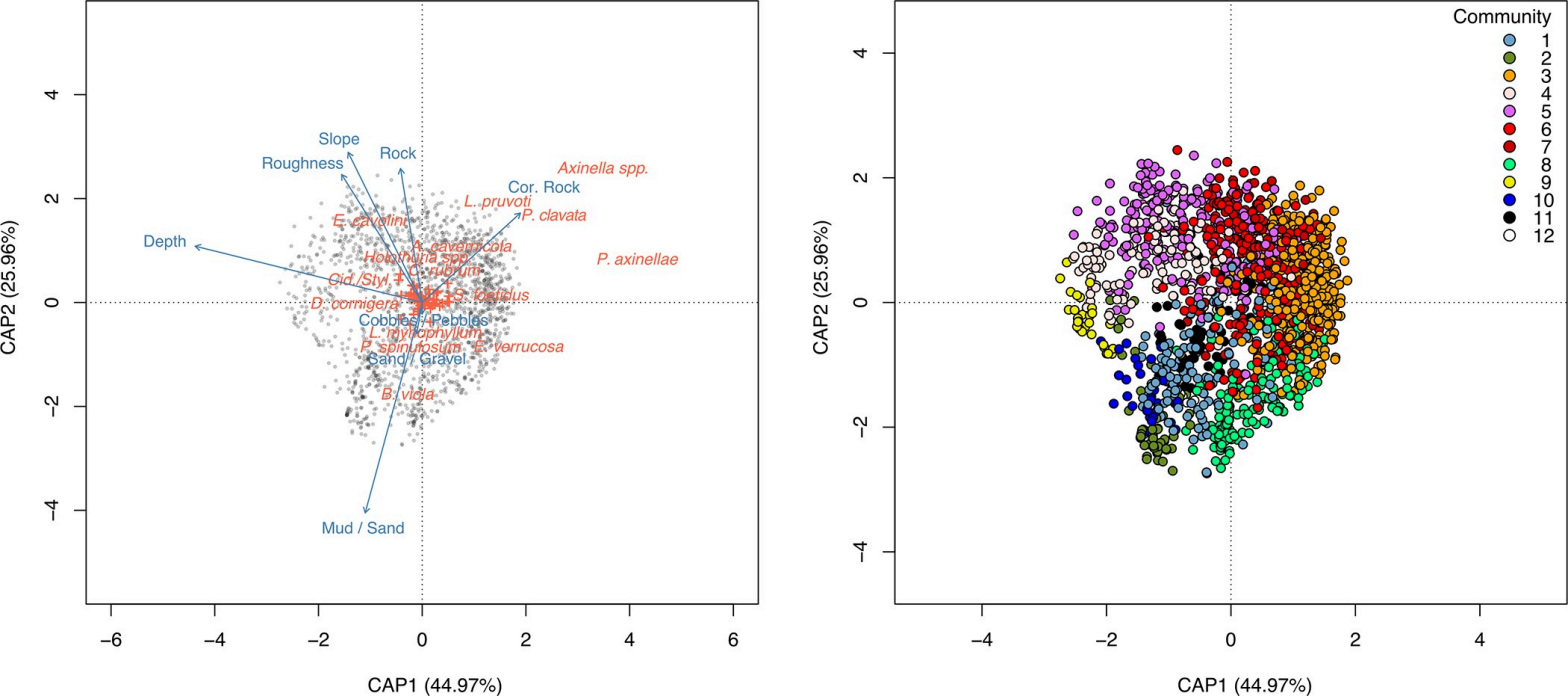
Distance-based redundancy analysis. Results of the dbRDA performed over Bray-Curtis dissimilarity matrix of square-root transformed density data. Samples are represented as grey or colored dots, environmental variables as solid lines with arrows and species as orange crosses. Names of the species with the highest scores are also shown in the biplot.

Based on the dbRDA, the selected factors explained 9.5% of the total variability observed in the samples regarding species composition, with the first two axes explaining almost 71% of the constrained variance (CAP1: 45%, CAP2: 26%; Fig 9). Depth was the best predictor among all used factors, with the substrates “Mud/Sand” and “Rock”, seabed roughness and slope also important in explaining the spatial distribution of the benthic fauna. The biplot shows that in shallow areas, the complex *P*. *axinellae*-*Axinella* spp. was dominant on coralligenous outcrops, together with *P*. *clavata* and *L*. *pruvoti*, while detritic flat bottoms were characterized by *E*. *verrucosa*, serpulids, keratose sponges and the bryozoan *Turbicellepora avicularis* (Hincks, 1860). At intermediate depths, *A*. *cavernicola*, *C*. *rubrum* and *Haliclona poecillastroides* (Vacelet, 1969) were the most representative species, although muddy and sandy bottoms were dominated by *B*. *viola*, *P*. *spinulosum* and *L*. *myriophyllum*. At greater depths, outcropping rocks were generally dominated by *E*. *cavolini*, serpulids and holothurians.

### Rare and low-density communities

**Fig 10:**
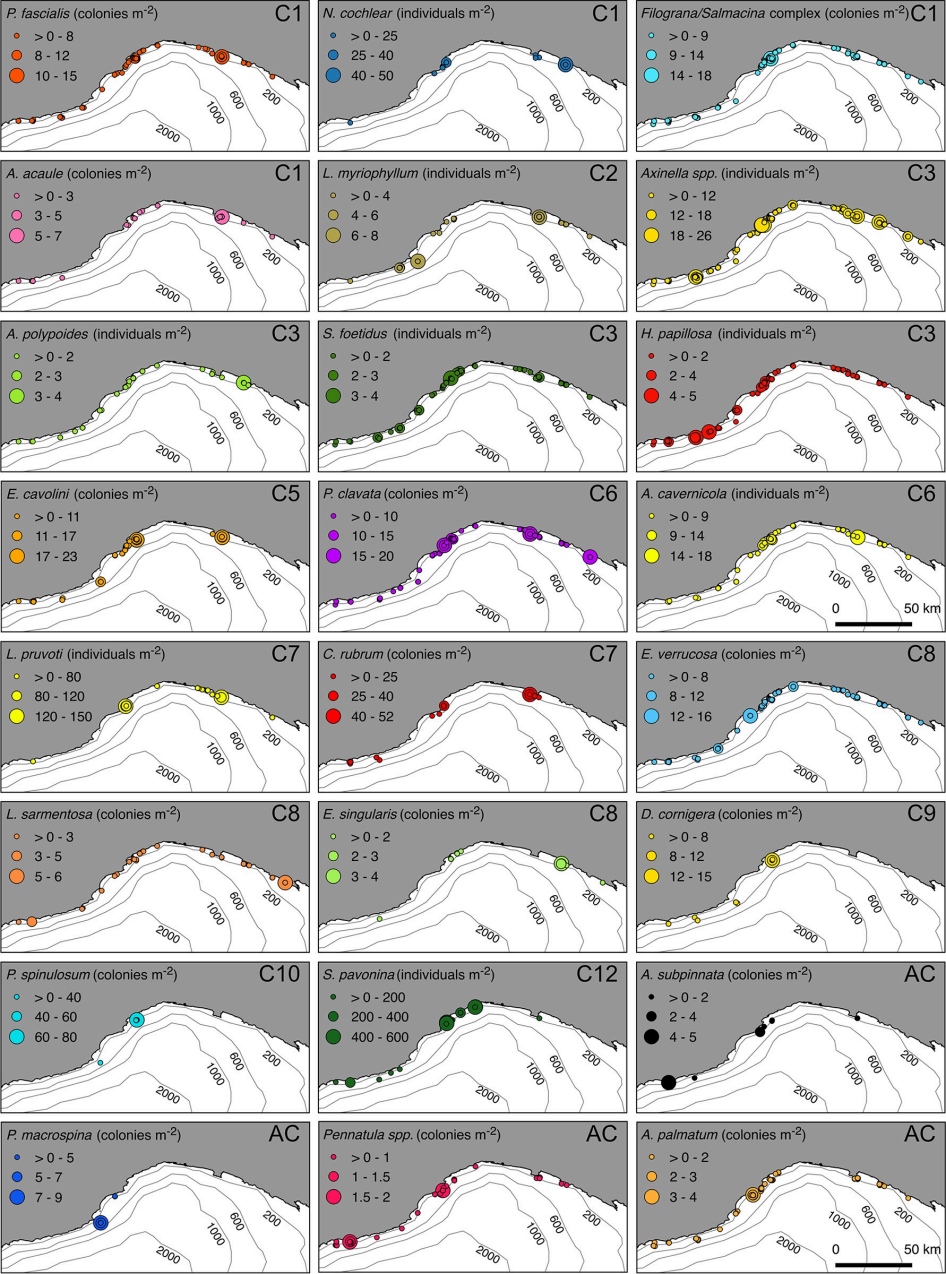
Distribution maps of the main habitat-forming species. Spatial distribution of 24 invertebrate species that are considered key structural organisms of the benthic communities identified in the cluster analysis. Codes C1 to C12 refer to the community the species belongs to, and AC to the additional communities.

The density and distribution of the main structuring species encountered along the continental shelf of Liguria is provided in Fig 10. Among these, four conspicuous cnidarians were depicted as rare or forming low-density communities, namely the black coral *Antipathella subpinnata* (Ellis & Solander, 1786), the gorgonian *Paramuricea macrospina* (Koch, 1882), the pennatulacean *Pennatula* spp., and the soft coral *A*. *palmatum*. Six forests of *A*. *subpinnata* occurred between 60 and 100 m depth, on horizontal or sloping rocks (Fig 7C). *A*. *subpinnata* was found in the sites of Santo Stefano, Finale Ligure, Mantice Shoal and Punta del Faro, reaching maximum densities of 4.4 col·m^-2^ in Bordighera. Other antipatharians were observed in the site of Mele Cape, namely *Leiopathes glaberrima* (Esper, 1788) (190–210 m depth) and *Parantipathes larix* (Esper, 1788) (114–212 m depth) (data not shown in map). The site of Mele Cape hosted the best developed *P*. *macrospina* aggregation (up to 9 col·m^-2^), occurring on rocky bottoms at 100 m depth, where it formed mixed forests with *P*. *clavata* and *E*. *cavolini* (Fig 7D). A shallower population of *P*. *macrospina* (75–80 m) was also reported from the site of Finale Ligure.

Soft bottoms, covered by mud and fine sands, appeared widespread within the Ligurian continental shelf. In those areas, pennatulaceans and soft corals were characterizing species, always observed with low densities (Fig 7E and 7F). These organisms were found scattered along the entire study area, with higher densities in the western sector, especially in the proximity of canyons heads. *Pennatula* spp. (Fig 7E) were observed between 40 and 130 m depth, with a maximum density of 3 col·m^-2^ in the sites of Bordighera and Noli Cape. The soft coral *A*. *palmatum* (Fig 7F) occurred between 40 and 140 m, with maximum densities of up to 3 col·m^-2^ in the site of Finale Ligure.

## Discussion

### Megabenthic diversity

The recent development of marine technology allows direct observation of deep-sea ecosystems with great detail, providing invaluable data on their composition and main structuring species. In the Mediterranean basin, local or sub-regional studies characterizing the diversity of invertebrate megafauna based on video images have recently become popular [56,57,65,66], but investigations providing quantitative data over large geographical extents are still scarce, with only a few exceptions [56,57,65]. Our work represents one of the largest studies of mesophotic megabenthic communities identified using multivariate community-based analysis for the Mediterranean Sea, covering over 62 km of continental shelf and shelf break.

Overall, the twilight zone of the Ligurian Sea should be considered very rich due to the high number of megafauna species that it sustains. The explored area accounts for over 220 taxa, exceeding the almost 170 taxa found in Cap de Creus [57], the 118 found on the deep-shoals of Saint Eufemia Gulf in Calabria [67], and the around 70 reported in the Menorca Channel [56] and on the Seco de los Olivos Seamount [68]. The high number of species could be related to the large area sampled and the occurrence of deep coralligenous reefs and well-structured animal forests known to be hotspots of biodiversity [29,69]. A large percentage of the Ligurian species is represented by canopy-forming organisms, like massive/erect sponges, large hydroids, gorgonians and black corals, as well as species of smaller sizes, including bryozoans, alcyonaceans and sabellids. Most of the identified species are suspension feeders, a functional group that typically dominates at these depths in the Mediterranean Sea [56,57,65,66,67] and in all the communities herein described (with the only exception of Community 4, occurring on hard bottoms in all the investigated areas, and characterized by the complete absence of large structuring species). These complex, three-dimensional communities play a key role in the maintenance of the overall benthic diversity of the area and must be considered a fundamental link for energy transfer between the pelagic and the benthic ecosystem [30,33,34].

This study provides the most comprehensive characterization of the megabenthic communities of the mesophotic Ligurian deep continental shelf and shelf break to date. Until now, only a few, local biocoenotic characterizations had been carried out on these communities in this area [25,26,37]. Some of the aggregations identified here had previously been reported for the Ligurian Sea, but their geographical and bathymetrical distributions had never been investigated over such a large spatial extent. Major differences in the spatial distribution of the benthic diversity were detected along the study area, as previously reported [27], with the western sector displaying higher numbers of species and communities. These patterns might be explained by the particular morphology of its narrow continental shelf, incised by several canyons, that probably enhances upwelling processes and promotes habitat heterogeneity, with the presence of several shoals, vertical walls and deep banks [4,50,70]. Conversely, the homogeneity of the wide continental shelf and large terrigenous supply introduced by River Magra in the eastern sector [4,54,55] might explain the much lower diversity values recorded, as well as the highest abundance of species showing an affinity for silted environments, such as *Leptogorgia sarmentosa* (Esper, 1789) [71]. Site-specific differences in the benthic communities, moreover, could also be explained by local variations in the trophic conditions due to the high urbanisation of the coast, the presence of black water discharge without sewage systems, the amount of river inputs, as well as specific hydrological patterns [72]. In addition, Casella et al. [50] identified mesoscale eddies, which may be responsible for upwelling currents contributing to the increase in the amount of food available in the water column. These eddies occur in the western sector of the Ligurian Sea, and correspond to the densest aggregations of *S*. *foetidus*, *H*. *papillosa* and *B*. *viola*.

### Gorgonian forests

The most diverse communities identified over the Ligurian mesophotic hardgrounds were those characterized by the presence of gorgonian species. It is widely known that these organisms play a major structuring role, especially between 40 and 200 m depth [28]. In the case of the Ligurian Sea, the octocorals *E*. *cavolini*, *P*. *clavata* and *E*. *verrucosa* constitute the dominant structuring species of three different communities (5, 6 and 8, respectively), each of them displaying a specific distribution pattern related to a particular depth range, slope and substrate type. Maximum gorgonian densities were in line with those reported in other areas of the Mediterranean Sea [58,73,74,75,76]. *E*. *verrucosa* is the less studied gorgonian species of the three, its presence being considered sporadic in the north-western basin [73]. Thus, the occurrence of large forests of this species in the Ligurian Sea is noteworthy. The observation of a few colonies of the structuring zoanthid *Savalia savaglia* (Bertoloni, 1819) within two of the explored *P*. *clavata* forests is also significant, suggesting that the occurrence of this species is rare and that the mesophotic forest of Portofino remains the largest known so far for this region [25].

It is known that the Maledetti Shoal (at mesophotic depths) and the Portofino Promontory (in shallow-waters) host the most important red coral populations (Community 7) of the Ligurian Sea, once subject to harvesting pressure and now slowly recovering [77,78,79]. The densities of *C*. *rubrum* reported here appeared consistently lower than other studies conducted in these same areas or in other sites of the Tyrrhenian and Ligurian Sea [78,80,81]. Our low values could relate to the size of the SU employed, since surveys of red coral generally use samples between 0.04 and 1.25 m^2^ in order to investigate their patchy distribution. It is likely that the 5 m^2^ SU adopted in this study is too large to detect small-scale differences in the density of this species, which usually grows in narrow crevices and interstices separated by areas with no specimens.

### Sponge grounds

One of the most interesting findings of this study is the presence of dense aggregations of the large keratose sponge *S*. *foetidus* (Community 3), with densities never reported before from the western Mediterranean Sea. Keratose sponge grounds are considered rare in the Mediterranean Sea, with only few examples from the eastern basin [82,83,84] and facies of massive/erect sponges reported for the Ligurian Sea [27]. Recent surveys conducted in 2018 (authors personal communication) expanded the range of distribution of these sponge grounds, which were also reported in the area of Sanremo (western sector), suggesting a vast occurrence of these biocoenoses in the NW Mediterranean basin and supporting a significant structural and ecological role of keratose sponges.

Overall, the small sponges *Axinella* spp., very common along the whole Mediterranean basin [85,86], were the most abundant species within Community 3. These sponges have been observed in assemblages dominated by *Axinella damicornis* (Esper, 1794) and *Axinella verrucosa* (Esper, 1794) together with other semi-sciaphilous organisms dwelling on horizontal, coralligenous, rocky outcrops or maërl/rhodolite beds from the inner continental shelf, at depths of 50 to 100 m (Menorca Channel) [87]. In the Ligurian Sea, this sponge community occurs on a similar substrate and slope, but mostly concentrated between 40 and 60 m depth, which might be explained by the turbid waters of Liguria compared to those of the Menorca Channel. The distribution and density data reported here, as well as the commonly observed symbiosis of *Axinella* spp. with the anthozoan *P*. *axinellae*, supports the fact that sponges act as ecosystem engineers at a smaller scale [88,89,90]. The large water masses moved by the aquifer systems of the sponges could be enhancing the development of a rich array of associated species and epibionts [31,91]. The ascidian *H*. *papillosa* is often present within this community, also enhancing the water fluxes by active filter-feeding [92]. Furthermore, from a structural point of view, other large sponges contributed considerably to increase the three-dimensionality of these sponge grounds, with examples such as the vase-like *S*. *lamella* and the arborescent *A*. *polypoides*. Indeed, this community presents the highest average diversity per sample. In contrast, sponge aggregations of Community 11 dominated by *H*. cf. *mediterranea* were characterized by low diversity values. They are found only in the site of Santo Stefano, characterized by flat, detritic bottoms. A similar aggregation was reported from the inner continental shelf of the Menorca Channel [87], where the sponge *Haliclona (Reniera) mediterranea* Griessinger, 1971 occurred on horizontal maërl beds between 60 and 110 m depth.

### Soft bottom communities

The remaining aggregations mainly developed on detritic and other soft-bottom areas, with only one community displaying a wide geographical and bathymetrical distribution (Community 1, bryozoan beds). In the Mediterranean Sea, bryozoans are important structuring species, considered the most important animal group among coralligenous reefs builders [93,94]. In Liguria, monospecific patches of the bryozoan *P*. *fascialis* have been previously reported from Tinetto Island and Alassio, at shallow depths (10–25 m) [14,93,95,96]. Due to their fragility, their occurrence, especially on horizontal, detritic bottoms, can be considered an indicator of environmental stability, especially with reference to shallow-water coastal trawling activities.

The remaining soft-bottom communities showed more restrictive preferences in terms of environmental requirements. Until now, *L*. *myriophyllum* forests were only reported near Imperia, Portofino and Monterosso [5,26,97,98]. Our findings indicate that the occurrence of these forests is broader, including eight new sites (Fig 10), where dense aggregations were found (1.57 col·m^-2^). These observations support the distribution of these forests on muddy bottoms mixed with biogenic mineral debris at the base of rocky cliffs, characterized by a high terrigenous supply.

Similar conclusions can be drawn for the three aggregations of *D*. *cornigera* reported here, which develop on soft-detritic bottoms near rocky shoals. *D*. *cornigera* aggregations were also reported from the canyons of the Gulf of Lions [65], but only the Ligurian population and a smaller one reported on the Amendolara Seamount [99] distinctively develop on soft bottoms. The aggregation lying at the base of the Mantice Shoal [37] represents the largest and northernmost record of *D*. *cornigera* within the Mediterranean basin.

Regarding the soft coral *P*. *spinulosum* (Community 10), this species usually occurs on hard bottoms [100,101,102,103,104], reaching densities up to 30 col·m^-2^ [105]. Similar patches of the soft-coral *Nidalia studeri* (Koch, 1891) have been reported from gently sloping rocky outcrops in the Menorca Channel [56]. The meadows recorded near the Mantice Shoal occur on soft muddy bottoms, where *P*. *spinulosum* colonies settle on fine biogenic detritus and reach the highest abundance known for this species. Again, all these communities mainly occurred in the western sector of Liguria, where a more complex topography and peculiar hydrographic conditions enhance the amount of nutrients in the water.

Finally, habitats dominated by *B*. *viola* are only known from the Canary Islands [106]. Sabellid beds resembling those of Community 12 have also been reported from a detrital bottom at 90 m depth in front of Cap Ferrat (western Ligurian Sea) during the MEDSEASCAN surveys [107]. In that case, the dominant species identified was *Sabella pavonina* Savigny, 1822 and the exceptional abundance of annelids was explained by the high levels of organic input derived from black water discharges nearby. Within Liguria, all the sites reporting dense fields of *B*. *viola*, including some additional recent observations near Sanremo (author’s personal communication) are close to highly urbanized coasts where polluted water discharges are known to occur.

### Rare and low-density communities

Multivariate analyses did not detect certain aggregations that could be visually identified directly from the video images, mainly due to low density values of the key structuring species. This is the case for the largest part of the soft bottoms located between 40 and 140 m depth, which were dominated by the soft coral *A*. *palmatum* and the sea pen *Pennatula* spp. Both species showed similar densities to those reported in other shelf areas of the Mediterranean, such as Cap de Creus [57]. In the case of *Pennatula* spp., the density observed in the Ligurian Sea appears three orders of magnitude higher than that observed in the Adriatic and Ionian Seas, where abundance data are derived from trawling bycatch. Differences in the quantification method could underestimate local patches of very high densities, which can only be accurately determined using video images [108,109,110]. Despite the ecological role of these soft-bottom engineers, their resilience to trawling is far from being fully understood and hence their occurrence is extremely relevant for the conservation of areas subjected to fishing activities [111].

Aggregations of black corals were not identified as distinct communities either, although three species of large antipatharians were observed in the images. *A*. *subpinnata* represented the most common black coral within the basin at mesophotic depths with a total of six aggregations, with two additional populations found developing on top of wrecks [112]. In contrast, *L*. *glaberrima* and *P*. *larix* occurred only in one deep dive, supporting previous observations regarding the distribution of these two species primarily along the shelf break and the upper slope [113]. Like gorgonians, large antipatharians act as major structuring species in Mediterranean benthic habitats [24,28] and their presence must be considered significant for the Ligurian biodiversity. Given their slow growth rates, their vulnerability to human impacts and their key role as nursery areas [32], black corals are recognized as endangered species and the georeferenced records here reported provide essential information for their conservation.

The gorgonian *P*. *macrospina* has been widely reported from the western Mediterranean Sea [67,76], while records within the Ligurian Sea were scarce until now [114]. A small fragment was reported in the trawling discard from the Gulf of Genova by Rossi [18], and another specimen was described from the cliffs of Gallinara Island, below 50 m depth [7]. In the present study, neither *P*. *macrospina* nor *P*. *clavata* were observed near the Gallinara Island, and the two populations of *P*. *macrospina* described here were found in deeper areas, lying below 75 m in an ecological setting similar to that described in the rocky coral oases of the South Tyrrhenian Sea subjected to moderate silting [67].

### Concluding remarks

This study highlights the rich megabenthic communities that can be found on the Ligurian deep continental shelf, filling an important knowledge gap about the Mediterranean mesophotic benthic biodiversity. Additional research will be necessary in order to investigate the population structure of each community identified in this study, leading to a better ecological interpretation of such communities.

Marine animal forests have historically been recognized as vulnerable to numerous anthropogenic activities [28,115] and some protection measures are now implemented to protect these fragile ecosystems, including the creation of lists of protected species and habitats, the adoption of fishing restrictions and the delineation of fisheries restricted areas [116,117]. Within the area investigated in this study, a large effort must be devoted to the identification and quantification of the major pressures that threaten these ecosystems and to characterize their health status. In any case, knowledge about the diversity and distribution of structuring organisms represents the first and indispensable step to establish effective management measures for the conservation of complex marine ecosystems. The present study provides the first georeferenced database of the Ligurian mesophotic megabenthic species and communities, and managers and stakeholders should use this tool to identify ecologically important sites, eventually leading to the design of an effective network of marine protected areas, aiming to preserve the diversity of megabenthic communities that dwell in Ligurian waters.

## Supporting information

S1 Table Technical information for the 80 ROV dives carried out on the ligurian continental shelf and shelf break. (DOCX)

S2 Table Main environmental features of the 80 ROV dives carried out on the ligurian continental shelf and shelf break. (DOCX)

S3 Table Comprehensive list of the species identified in this study, with their density values and occupancy. (DOCX)

S4 Table Biological features of the 80 ROV dives carried out on the ligurian continental shelf and shelf break. (DOCX)
