# Supplementary material for: Megabenthic communities of the Ligurian deep continental shelf and shelf break (NW Mediterranean Sea)
Source: PLoS One. 2019 Oct 17;14(10):e0223949. doi: 10.1371/journal.pone.0223949 (PMC6797210; doi:10.1371/journal.pone.0223949)
Supplement: S1 Table — (DOCX) [file pone.0223949.s001.docx]

**S1 Table. Technical information for the 80 ROV dives carried out on the Ligurian continental shelf and shelf break.**

| **Dive** | **Date** | **Area** | | **Site** | **ROV** | **Start position** | | **End position** | | **Depth (m)** | | **Length (m)** | **Nº SU** | **% valid SU** |
| --- | --- | --- | --- | --- | --- | --- | --- | --- | --- | --- | --- | --- | --- | --- |
|  |  |  |  |  |  | **Lat.**  **N** | **Long E.** | **Lat.**  **N** | **Long. E** | **Start** | **End** |  |  |  |
| A01 | 01/09/16 | | Ventimiglia | Mortola Cape | PIII | 7.552 | 43.743 | 7.555 | 43.742 | 89 | 85 | 545 | 54 | 87 |
| A02 | 01/09/16 | | Ventimiglia | Mortola Cape | PIII | 7.564 | 43.738 | 7.567 | 43.739 | 86 | 82 | 373 | 37 | 89 |
| A03 | 01/09/16 | | Ventimiglia | Mortola Cape | PIII | 7.563 | 43.769 | 7.559 | 43.772 | 45 | 30 | 1150 | 115 | 48 |
| A04 | 02/09/16 | | Ventimiglia | Bordighera | PIII | 7.675 | 43.770 | 7.682 | 43.766 | 39 | 98 | 1595 | 159 | 62 |
| A05 | 02/09/16 | | Ventimiglia | Bordighera | PIII | 7.680 | 43.774 | 7.682 | 43.774 | 69 | 55 | 186 | 18 | 44 |
| A06 | 02/09/16 | | Ventimiglia | Bordighera | PIII | 7.670 | 43.770 | 7.671 | 43.772 | 76 | 43 | 974 | 97 | 48 |
| A07 | 07/06/12 | | Ventimiglia | Bordighera | PIII | 7.682 | 43.780 | 7.680 | 43.773 | 38 | 75 | 1797 | 179 | 39 |
| A08 | 09/06/12 | | Ventimiglia | Besughi Shoal | PIII | 7.678 | 43.754 | 7.679 | 43.754 | 120 | 190 | 230 | 23 | 48 |
| B01 | 07/06/12 | | Imperia | Gobbe del Cammello | PIII | 7.918 | 43.784 | 7.916 | 43.781 | 96 | 156 | 778 | 77 | 39 |
| B02 | 12/08/15 | | Imperia | Santo Stefano | PIII | 7.920 | 43.796 | 7.912 | 43.792 | 63 | 58 | 874 | 87 | 57 |
| B03 | 12/08/15 | | Imperia | Santo Stefano | PIII | 7.907 | 43.806 | 7.897 | 43.801 | 46 | 66 | 1625 | 162 | 28 |
| B04 | 13/08/15 | | Imperia | Santo Stefano | PIII | 7.892 | 43.809 | 7.892 | 43.803 | 87 | 87 | 841 | 84 | 17 |
| B05 | 13/08/15 | | Imperia | Porto Maurizio | PIII | 8.010 | 43.848 | 8.011 | 43.847 | 47 | 48 | 789 | 78 | 15 |
| B06 | 13/08/15 | | Imperia | Porto Maurizio | PIII | 8.023 | 43.851 | 8.030 | 43.851 | 36 | 37 | 794 | 79 | 28 |
| B07 | 14/08/15 | | Imperia | Diano Marina | PIII | 8.086 | 43.882 | 8.080 | 43.875 | 49 | 53 | 1761 | 176 | 41 |
| B08 | 14/08/15 | | Imperia | Diano Marina | PIII | 8.096 | 43.878 | 8.097 | 43.876 | 65 | 70 | 273 | 27 | 78 |
| C01 | 06/06/12 | | Alassio | Mele Cape | PIII | 8.230 | 43.930 | 8.231 | 43.929 | 106 | 135 | 754 | 75 | 68 |
| C02 | 06/06/12 | | Alassio | Mele Cape | PIII | 8.240 | 43.923 | 8.243 | 43.919 | 179 | 212 | 674 | 67 | 30 |
| C03 | 16/02/18 | | Alassio | Gallinara Island | BR2 | 8.226 | 44.009 | 8.226 | 44.004 | 80 | 89 | 736 | 73 | 95 |
| C04 | 17/04/18 | | Alassio | Gallinara Island | BR2 | 8.223 | 44.022 | 8.229 | 44.024 | 40 | 39 | 756 | 75 | 85 |
| C05 | 15/02/18 | | Alassio | Gallinara Island | BR2 | 8.240 | 44.023 | 8.240 | 44.024 | 66 | 59 | 474 | 47 | 94 |
| C06 | 14/02/18 | | Alassio | Gallinara Island | BR2 | 8.247 | 44.026 | 8.244 | 44.028 | 60 | 55 | 390 | 39 | 100 |
| D01 | 17/04/18 | | Finale Ligure | Pora Canyon | BR2 | 8.347 | 44.144 | 8.346 | 44.147 | 78 | 76 | 524 | 52 | 96 |
| D02 | 17/04/18 | | Finale Ligure | Pora Canyon | BR2 | 8.362 | 44.157 | 8.354 | 44.155 | 88 | 78 | 822 | 82 | 93 |
| D03 | 17/04/18 | | Finale Ligure | Pora Canyon | BR2 | 8.364 | 44.156 | 8.366 | 44.157 | 92 | 82 | 587 | 58 | 97 |
| D04 | 18/04/18 | | Finale Ligure | Pora Canyon | BR2 | 8.369 | 44.152 | 8.365 | 44.157 | 92 | 85 | 639 | 63 | 90 |
| D05 | 06/06/12 | | Finale Ligure | Lua Canyon | PIII | 8.409 | 44.161 | 8.405 | 44.161 | 96 | 138 | 752 | 75 | 75 |
| D06 | 18/04/18 | | Finale Ligure | Noli Cape | BR2 | 8.426 | 44.185 | 8.426 | 44.181 | 93 | 85 | 580 | 58 | 79 |
| D07 | 21/04/18 | | Finale Ligure | Noli Cape | BR2 | 8.433 | 44.188 | 8.428 | 44.187 | 106 | 103 | 703 | 70 | 81 |
| D08 | 10/08/15 | | Finale Ligure | Noli Canyon | PIII | 8.432 | 44.201 | 8.435 | 44.211 | 71 | 72 | 1862 | 186 | 38 |
| E01 | 10/08/15 | | Savona | Maledetti Shoal | PIII | 8.434 | 44.222 | 8.437 | 44.224 | 53 | 83 | 819 | 81 | 46 |
| E02 | 03/09/16 | | Savona | Maledetti Shoal | PIII | 8.445 | 44.227 | 8.442 | 44.226 | 55 | 111 | 640 | 64 | 38 |
| E03 | 05/06/12 | | Savona | Corallone Shoal | PIII | 8.459 | 44.225 | 8.459 | 44.223 | 64 | 122 | 667 | 66 | 45 |
| E04 | 09/08/15 | | Savona | Vado shoals | PIII | 8.458 | 44.248 | 8.463 | 44.247 | 39 | 76 | 692 | 69 | 70 |
| E05 | 09/08/15 | | Savona | Vado shoals | PIII | 8.466 | 44.253 | 8.465 | 44.251 | 63 | 69 | 373 | 37 | 59 |
| E06 | 09/08/15 | | Savona | Vado shoals | PIII | 8.465 | 44.261 | 8.463 | 44.260 | 64 | 45 | 843 | 84 | 62 |
| E07 | 05/06/12 | | Savona | Mantice Shoal | PIII | 8.522 | 44.270 | 8.521 | 44.267 | 105 | 151 | 1013 | 101 | 45 |
| E08 | 05/06/12 | | Savona | Mantice Shoal | PIII | 8.525 | 44.273 | 8.524 | 44.271 | 84 | 80 | 399 | 39 | 54 |
| E09 | 09/08/15 | | Savona | Mantice Shoal | PIII | 8.526 | 44.273 | 8.524 | 44.271 | 106 | 81 | 844 | 84 | 33 |
| E10 | 26/08/15 | | Savona | Mantice Shoal | PIII | 8.528 | 44.279 | 8.525 | 44.273 | 78 | 106 | 1243 | 124 | 31 |
| E11 | 03/09/16 | | Savona | Savona shoals | PIII | 8.524 | 44.280 | 8.523 | 44.275 | 73 | 79 | 1289 | 128 | 57 |
| E12 | 03/09/16 | | Savona | Savona shoals | PIII | 8.516 | 44.279 | 8.513 | 44.277 | 65 | 70 | 732 | 73 | 81 |
| E13 | 03/09/16 | | Savona | Savona shoals | PIII | 8.507 | 44.280 | 8.514 | 44.279 | 55 | 59 | 924 | 92 | 51 |
| E14 | 03/09/16 | | Savona | Savona shoals | PIII | 8.500 | 44.287 | 8.502 | 44.282 | 56 | 48 | 987 | 98 | 68 |
| F01 | 04/09/16 | | West Genova | Celle Ligure | PIII | 8.551 | 44.331 | 8.551 | 44.327 | 44 | 52 | 1154 | 115 | 58 |
| F02 | 08/08/15 | | West Genova | Varazze | PIII | 8.573 | 44.340 | 8.574 | 44.342 | 47 | 44 | 628 | 62 | 68 |
| F03 | 08/08/15 | | West Genova | Varazze | PIII | 8.578 | 44.338 | 8.575 | 44.336 | 61 | 62 | 559 | 55 | 93 |
| F04 | 08/08/15 | | West Genova | Varazze | PIII | 8.582 | 44.347 | 8.582 | 44.346 | 40 | 47 | 839 | 83 | 100 |
| F05 | 08/08/15 | | West Genova | Arenzano | PIII | 8.695 | 44.387 | 8.692 | 44.387 | 41 | 43 | 848 | 84 | 62 |
| F06 | 08/08/15 | | West Genova | Arenzano | PIII | 8.700 | 44.385 | 8.697 | 44.383 | 62 | 62 | 536 | 53 | 91 |
| G01 | 06/08/15 | | East Genova | Nervi | PIII | 9.032 | 44.364 | 9.027 | 44.364 | 52 | 56 | 497 | 49 | 69 |
| G02 | 06/08/15 | | East Genova | Nervi | PIII | 9.034 | 44.369 | 9.023 | 44.371 | 34 | 34 | 1285 | 128 | 100 |
| G03 | 06/08/15 | | East Genova | Bogliasco | PIII | 9.066 | 44.358 | 9.059 | 44.360 | 57 | 55 | 839 | 83 | 66 |
| G04 | 05/08/15 | | East Genova | Sori | PIII | 9.089 | 44.361 | 9.083 | 44.360 | 35 | 37 | 614 | 61 | 77 |
| G05 | 05/08/15 | | East Genova | Sori | PIII | 9.100 | 44.353 | 9.108 | 44.354 | 53 | 43 | 958 | 95 | 60 |
| H01 | 04/09/16 | | Portofino | Punta Chiappa | PIII | 9.148 | 44.337 | 9.149 | 44.334 | 34 | 34 | 625 | 62 | 87 |
| H02 | 03/06/12 | | Portofino | Isuela | PIII | 9.144 | 44.320 | 9.144 | 44.320 | 29 | 31 | 245 | 24 | 50 |
| H03 | 05/09/16 | | Portofino | Isuela | PIII | 9.145 | 44.320 | 9.143 | 44.318 | 52 | 73 | 939 | 93 | 69 |
| H04 | 06/09/16 | | Portofino | Cala degli Inglesi | PIII | 9.190 | 44.305 | 9.185 | 44.307 | 73 | 50 | 806 | 80 | 69 |
| H05 | 27/08/15 | | Portofino | San Gorgio shoal | PIII | 9.191 | 44.283 | 9.191 | 44.283 | 104 | 104 | 255 | 25 | 44 |
| H06 | 27/08/15 | | Portofino | San Gorgio shoal | PIII | 9.197 | 44.294 | 9.195 | 44.287 | 96 | 104 | 1098 | 109 | 71 |
| H07 | 03/06/12 | | Portofino | San Gorgio shoal | PIII | 9.205 | 44.292 | 9.207 | 44.293 | 98 | 92 | 544 | 54 | 59 |
| H08 | 02/06/12 | | Portofino | Punta del Faro | PIII | 9.221 | 44.287 | 9.223 | 44.292 | 100 | 76 | 923 | 92 | 67 |
| H09 | 25/08/16 | | Portofino | Punta del Faro | PIII | 9.222 | 44.287 | 9.220 | 44.286 | 101 | 104 | 503 | 50 | 72 |
| H10 | 02/06/12 | | Portofino | Punta del Faro | PIII | 9.223 | 44.292 | 9.217 | 44.297 | 75 | 57 | 1494 | 149 | 38 |
| H11 | 25/08/16 | | Portofino | Punta del Faro | PIII | 9.219 | 44.293 | 9.222 | 44.291 | 83 | 83 | 722 | 72 | 89 |
| H12 | 25/08/16 | | Portofino | Punta del Faro | PIII | 9.223 | 44.295 | 9.217 | 44.297 | 86 | 63 | 747 | 74 | 86 |
| H13 | 25/08/16 | | Portofino | Punta del Faro | PIII | 9.220 | 44.298 | 9.218 | 44.297 | 61 | 59 | 908 | 90 | 39 |
| I01 | 03/08/15 | | Sestri Levante | Punta Manara | PIII | 9.400 | 44.246 | 9.403 | 44.245 | 46 | 56 | 676 | 67 | 94 |
| I02 | 03/08/15 | | Sestri Levante | Punta Manara | PIII | 9.397 | 44.246 | 9.401 | 44.242 | 62 | 64 | 768 | 76 | 88 |
| I03 | 03/08/15 | | Sestri Levante | Punta Manara | PIII | 9.404 | 44.245 | 9.402 | 44.240 | 58 | 74 | 586 | 58 | 69 |
| I04 | 23/08/16 | | Sestri Levante | Punta Manara | PIII | 9.401 | 44.242 | 9.398 | 44.245 | 65 | 62 | 625 | 62 | 47 |
| I05 | 03/08/15 | | Sestri Levante | Punta Manara | PIII | 9.399 | 44.239 | 9.401 | 44.236 | 81 | 85 | 562 | 56 | 84 |
| I06 | 03/08/15 | | Sestri Levante | Punta Baffe | PIII | 9.436 | 44.229 | 9.439 | 44.230 | 73 | 66 | 512 | 51 | 86 |
| I07 | 03/08/15 | | Sestri Levante | Punta Baffe | PIII | 9.439 | 44.231 | 9.444 | 44.230 | 56 | 58 | 674 | 67 | 87 |
| I08 | 03/08/15 | | Sestri Levante | Punta Baffe | PIII | 9.444 | 44.234 | 9.441 | 44.236 | 37 | 33 | 383 | 38 | 76 |
| J01 | 02/09/16 | | Cinque Terre | Punta Mesco | PIII | 9.631 | 44.133 | 9.635 | 44.132 | 35 | 48 | 597 | 59 | 42 |
| J02 | 23/08/16 | | Cinque Terre | Punta Mesco | PIII | 9.635 | 44.130 | 9.636 | 44.131 | 51 | 47 | 665 | 66 | 21 |
| J03 | 02/09/16 | | Cinque Terre | Corniglia | PIII | 9.692 | 44.112 | 9.689 | 44.112 | 40 | 42 | 621 | 62 | 73 |
| J04 | 23/08/16 | | Cinque Terre | Punta Montenero | PIII | 9.741 | 44.090 | 9.738 | 44.090 | 29 | 35 | 539 | 53 | 30 |

GPS positions are provided in decimal degrees. The different areas explored can be identified in Fig. 1. PIII: ROV Pollux III; BR2: BlueROV2; SU: sampling unit.
