# Supplementary material for: Megabenthic communities of the Ligurian deep continental shelf and shelf break (NW Mediterranean Sea)
Source: PLoS One. 2019 Oct 17;14(10):e0223949. doi: 10.1371/journal.pone.0223949 (PMC6797210; doi:10.1371/journal.pone.0223949)
Supplement: S2 Table — (DOCX) [file pone.0223949.s002.docx]

**S2 Table. Main environmental features of the 80 ROV dives carried out on the Ligurian continental shelf and shelf break.**

| **Dive** | **Depth (m)** | | **Substrate type (%)** | | | | | **Slope (deg)** | | **Roughness** | |
| --- | --- | --- | --- | --- | --- | --- | --- | --- | --- | --- | --- |
|  | **Min** | **Max** | **Mud/Sand** | **Sand/Gravel** | **Cobbl/Pebbl** | **Rock** | **Cor. Rock** | **Min** | **Max** | **Min** | **Max** |
| A01 | 81 | 89 | 96 |  |  | 4 |  | 0.0 | 16.6 | 0.02 | 1.61 |
| A02 | 82 | 86 | 100 |  |  |  |  | 1.1 | 9.5 | 0.09 | 0.84 |
| A03 | 28 | 47 | 55 | 1 |  | 28 | 17 | 0.3 | 34.8 | 0.03 | 3.37 |
| A04 | 39 | 100 | 47 |  |  | 44 | 8 | 0.0 | 50.8 | 0.00 | 5.58 |
| A05 | 53 | 69 | 79 |  |  | 21 |  | 13.8 | 57.6 | 1.17 | 7.37 |
| A06 | 42 | 99 | 53 |  | 11 | 36 | 1 | 0.5 | 48.2 | 0.06 | 6.21 |
| A07 | 38 | 80 | 63 |  |  | 35 |  | 4.8 | 15.6 | 0.05 | 7.36 |
| A08 | 128 | 200 | 3 |  |  | 97 |  | 30.0 | 45.0 | 0.21 | 6.18 |
| B01 | 88 | 154 | 13 | 2 | 11 | 74 |  | 8.7 | 55.0 | 0.03 | 3.02 |
| B02 | 52 | 63 | 14 | 55 |  | 21 | 10 | 0.1 | 28.5 | 0.02 | 1.19 |
| B03 | 45 | 67 | 1 | 74 |  | 10 | 15 | 0.2 | 55.1 | 0.01 | 2.97 |
| B04 | 85 | 92 | 14 |  |  | 86 |  | 0.9 | 59.6 | 0.05 | 4.38 |
| B05 | 45 | 52 | 42 |  |  | 8 | 50 | 0.3 | 42.8 | 0.02 | 2.02 |
| B06 | 33 | 39 | 3 | 69 |  | 12 | 16 | 0.1 | 34.8 | 0.02 | 2.38 |
| B07 | 49 | 54 | 43 |  |  | 10 | 47 | 0.0 | 47.1 | 0.00 | 2.54 |
| B08 | 65 | 70 | 99 |  |  | 1 |  | 0.2 | 17.1 | 0.02 | 0.83 |
| C01 | 99 | 135 | 4 |  |  | 96 |  | 30.0 | 45.0 | 0.05 | 6.39 |
| C02 | 190 | 216 | 34 |  |  | 66 |  | 9.6 | 83.0 | 0.12 | 12.03 |
| C03 | 80 | 87 | 95 |  |  | 5 |  | 1.5 | 18.1 | 0.19 | 1.62 |
| C04 | 28 | 40 | 74 |  |  | 4 | 22 | 4.9 | 27.1 | 0.44 | 2.72 |
| C05 | 58 | 66 | 62 |  |  | 38 |  | 0.7 | 35.8 | 0.12 | 3.62 |
| C06 | 55 | 60 | 100 |  |  |  |  | 0.2 | 6.5 | 0.04 | 0.61 |
| D01 | 74 | 89 | 40 |  |  | 60 |  | 8.1 | 57.3 | 0.77 | 7.86 |
| D02 | 75 | 109 | 100 |  |  |  |  | 0.6 | 23.9 | 0.18 | 2.3 |
| D03 | 78 | 92 | 80 |  |  | 18 |  | 0.6 | 50.5 | 0.08 | 5.71 |
| D04 | 84 | 96 | 100 |  |  |  |  | 1.1 | 19.8 | 0.12 | 2.36 |
| D05 | 91 | 129 | 15 |  |  | 85 |  | 11.4 | 75.0 | 0.18 | 6.68 |
| D06 | 86 | 94 | 68 |  |  | 32 |  | 0.9 | 33.1 | 0.13 | 4.67 |
| D07 | 93 | 108 | 53 |  |  | 47 |  | 2.6 | 70.0 | 0.38 | 16.1 |
| D08 | 71 | 141 | 90 |  |  | 10 |  | 3.9 | 15.6 | 0.05 | 5.15 |
| E01 | 51 | 85 | 41 |  |  | 59 |  | 1.1 | 79.4 | 0.08 | 11.1 |
| E02 | 54 | 111 | 8 |  |  | 91 | 1 | 8.2 | 85.0 | 0.05 | 12.5 |
| E03 | 65 | 110 | 59 |  |  | 41 |  | 9.6 | 38.0 | 0.03 | 8.22 |
| E04 | 39 | 76 | 87 |  |  | 8 | 5 | 0.4 | 50.0 | 0.02 | 2.83 |
| E05 | 59 | 69 | 78 |  |  | 20 |  | 0.1 | 63.5 | 0.06 | 3.97 |
| E06 | 36 | 71 | 62 |  |  | 38 |  | 0.7 | 68.7 | 0.09 | 7.33 |
| E07 | 79 | 151 | 61 |  |  | 39 |  | 1.3 | 70.0 | 0.01 | 3.08 |
| E08 | 78 | 88 | 35 |  |  | 65 |  | 1.5 | 9.8 | 0.31 | 6.26 |
| E09 | 85 | 121 | 92 |  |  | 8 |  | 3.1 | 49.7 | 0.23 | 6.26 |
| E10 | 77 | 108 | 48 |  |  | 52 |  | 0.5 | 55.2 | 0.07 | 7.58 |
| E11 | 65 | 85 | 58 |  |  | 34 | 9 | 0.1 | 49.3 | 0.03 | 5.88 |
| E12 | 56 | 72 | 62 |  |  | 11 | 27 | 0.4 | 63.4 | 0.07 | 8.87 |
| E13 | 51 | 60 | 70 |  |  | 18 | 12 | 0.2 | 17.8 | 0.03 | 1.72 |
| E14 | 46 | 56 | 60 |  |  | 34 | 6 | 0.1 | 24.0 | 0.01 | 2.34 |
| F01 | 39 | 52 | 64 | 1 |  | 31 | 4 | 1.8 | 2.3 | 0.04 | 1.08 |
| F02 | 38 | 47 | 62 |  |  | 10 | 28 | 0.1 | 25.9 | 0.02 | 1.22 |
| F03 | 60 | 63 | 98 |  |  | 2 |  | 0.1 | 11.1 | 0.02 | 0.49 |
| F04 | 40 | 52 | 72 |  |  | 27 | 1 | 0.3 | 17.9 | 0.01 | 0.87 |
| F05 | 34 | 42 | 74 |  |  | 6 | 20 | 0.4 | 44.1 | 0.02 | 2.29 |
| F06 | 56 | 62 | 80 |  |  | 19 |  | 0.3 | 17.0 | 0.38 | 0.74 |
| G01 | 52 | 56 | 80 |  |  | 20 |  | 0.2 | 9.0 | 0.04 | 0.40 |
| G02 | 30 | 36 |  | 53 |  | 24 | 23 | 0.1 | 62.2 | 0.01 | 4.18 |
| G03 | 53 | 57 | 69 |  |  | 18 | 13 | 0.3 | 29.8 | 0.05 | 1.40 |
| G04 | 33 | 37 |  | 48 |  | 12 | 40 | 0.1 | 48.6 | 0.01 | 2.32 |
| G05 | 43 | 53 | 64 |  |  | 11 | 25 | 0.1 | 28.3 | 0.02 | 1.52 |
| H01 | 34 | 36 | 3 | 48 | 10 | 1 | 37 | 0.1 | 32.1 | 0.01 | 1.56 |
| H02 | 29 | 44 |  |  |  |  | 100 | 5.3 | 81.8 | 0.31 | 15.3 |
| H03 | 34 | 72 | 8 | 41 |  | 11 | 40 | 0.2 | 72.4 | 0.09 | 8.17 |
| H04 | 34 | 73 | 10 | 27 | 50 |  | 12 | 1.0 | 52.6 | 0.32 | 1.55 |
| H05 | 103 | 106 | 72 |  |  | 28 |  | 0.6 | 0.9 | 0.02 | 1.55 |
| H06 | 93 | 104 | 90 |  |  | 10 |  | 0.4 | 0.9 | 0.01 | 1.48 |
| H07 | 89 | 99 | 60 |  |  | 40 |  | 0.8 | 0.9 | 0.01 | 1.31 |
| H08 | 73 | 101 | 85 |  |  | 15 |  | 0.5 | 12.0 | 0.02 | 2.13 |
| H09 | 101 | 104 | 90 |  |  | 6 |  | 1.3 | 6.3 | 0.02 | 1.78 |
| H10 | 55 | 90 | 53 |  |  | 44 | 3 | 0.2 | 10.0 | 0.03 | 4.36 |
| H11 | 61 | 83 | 65 |  |  | 35 |  | 0.3 | 53.1 | 0.04 | 3.07 |
| H12 | 60 | 86 | 88 |  |  | 12 |  | 0.1 | 64.8 | 0.03 | 4.88 |
| H13 | 57 | 78 | 59 |  | 11 | 31 |  | 0.4 | 64.5 | 0.07 | 5.67 |
| I01 | 42 | 54 | 56 |  |  | 35 | 9 | 0.3 | 49.3 | 0.02 | 2.79 |
| I02 | 58 | 68 | 70 |  |  | 30 |  | 0.3 | 26.4 | 0.03 | 1.30 |
| I03 | 58 | 74 | 68 |  |  | 32 |  | 0.2 | 49.2 | 0.02 | 3.17 |
| I04 | 59 | 67 | 83 |  |  | 17 |  | 0.1 | 23.6 | 0.03 | 1.19 |
| I05 | 79 | 85 | 100 |  |  |  |  | 0.1 | 12.5 | 0.03 | 0.56 |
| I06 | 66 | 73 | 81 |  |  | 7 |  | 0.2 | 39.6 | 0.03 | 2.02 |
| I07 | 51 | 58 | 61 |  |  | 38 |  | 0.3 | 21.5 | 0.05 | 0.98 |
| I08 | 33 | 41 | 52 | 15 | 18 | 13 | 3 | 1.9 | 9.8 | 0.08 | 1.49 |
| J01 | 36 | 49 | 2 | 51 |  | 14 | 34 | 0.2 | 67.4 | 0.02 | 5.38 |
| J02 | 47 | 55 | 48 |  |  | 45 | 7 | 0.1 | 59.3 | 0.03 | 3.37 |
| J03 | 40 | 42 | 100 |  |  |  |  | 0.0 | 32.9 | 0.00 | 1.81 |
| J04 | 28 | 30 | 13 | 6 |  | 9 | 72 | 0.1 | 52.9 | 0.01 | 3.11 |

Areas A-J can be identified in Fig 1.
