# Supplementary material for: Megabenthic communities of the Ligurian deep continental shelf and shelf break (NW Mediterranean Sea)
Source: PLoS One. 2019 Oct 17;14(10):e0223949. doi: 10.1371/journal.pone.0223949 (PMC6797210; doi:10.1371/journal.pone.0223949)
Supplement: S3 Table — (DOCX) [file pone.0223949.s003.docx]

**S3 Table.** **Comprehensive list of the species identified in this study, with their abundance values and occupancy.**

| **Phylum** | **Subgroup** | **Species** | **Total nº of organisms** | **Max density (ind m²)** | **% of ROV dives** | **% of SU** |
| --- | --- | --- | --- | --- | --- | --- |
| Porifera | Calcarea | *Clathrina* sp. | 63 | 0.12 | 18.75 | 0.77 |
|  | Demospongiae | *Acanthella acuta* | 33 | 0.12 | 16.25 | 0.37 |
|  |  | *Agelas oroides* | 617 | 0.56 | 48.75 | 3.58 |
|  |  | *Aplysina cavernicola* | 5113 | 3.36 | 57.5 | 6.83 |
|  |  | *Axinella polypoides* | 362 | 0.76 | 32.5 | 2.83 |
|  |  | *Axinella* spp. | 34314 | 5.08 | 88.75 | 36.48 |
|  |  | *Calyx nicaeensis* | 5 | 0.08 | 3.75 | 0.08 |
|  |  | *Chondrilla nucula* | 3 | 0.04 | 1.25 | 0.03 |
|  |  | *Chondrosia reniformis* | 780 | 1.64 | 57.5 | 3.52 |
|  |  | *Cliona* spp. | 49 | 0.52 | 6.25 | 0.27 |
|  |  | *Crella* sp. | 273 | 0.32 | 37.5 | 3.34 |
|  |  | *Dysidea* sp. | 906 | 1.52 | 53.75 | 4.7 |
|  |  | *Hexadella racovitzai* | 747 | 0.44 | 53.75 | 5.39 |
|  |  | *Ircinia* sp. | 30 | 0.12 | 16.25 | 0.67 |
|  |  | *Pachastrella monilifera* | 94 | 0.36 | 3.75 | 0.32 |
|  |  | *Petrosia (Petrosia) ficiformis* | 936 | 2.52 | 27.5 | 2 |
|  |  | *Pleraplysilla spinifera* | 81 | 0.16 | 25 | 1.01 |
|  |  | *Poecillastra compressa* | 36 | 0.16 | 3.75 | 0.29 |
|  |  | *Polymastia* sp. | 10 | - | 1.25 | - |
|  |  | *Raspailia (raspailia) viminalis* | 6 | 0.16 | 1.25 | 0.03 |
|  |  | *Rhizaxinell*a sp. | 3 | 0.04 | 1.25 | 0.05 |
|  |  | *Sarcotragus foetidus* | 1933 | 0.64 | 67.5 | 10.62 |
|  |  | *Sarcotragus spinosulus* | 9 | 0.12 | 2.5 | 0.05 |
|  |  | *Spongia lamella* | 85 | 0.16 | 32.5 | 1.25 |
|  |  | Tedania anhelans | 2 | 0.04 | 1.25 | 0.05 |
|  |  | *Tethya* spp. | 3 | 0.04 | 3.75 | 0.03 |
|  |  | *Ulosa stuposa* | 21 | 0.12 | 7.5 | 0.16 |
|  |  | *Haliclona* cf. *mediterranea* | 2447 | 3.08 | 16.25 | 2.46 |
|  |  | *Haliclona* sp. 1 | 112 | 0.12 | 22.5 | 0.64 |
|  |  | *Haliclona* sp. 2 | 77 | 0.28 | 22.5 | 0.72 |
|  |  | *Haliclona* sp. 3 | 2 | 0.04 | 1.25 | 0.03 |
|  |  | Keratosa sp. 1 | 242 | 0.48 | 42.5 | 2.8 |
|  |  | Keratosa sp. 3 | 196 | 0.28 | 26.25 | 2 |
|  |  | Keratosa sp. 4 | 39 | 0.08 | 18.75 | 0.45 |
|  |  | Keratosa sp. 5 | 176 | 0.24 | 26.25 | 1.73 |
|  |  | Keratosa sp. 8 | 18 | 0.08 | 15 | 0.24 |
|  |  | Keratosa sp. 9 | 11 | 0.16 | 5 | 0.11 |
|  |  | Porifera sp. 01 | 14 | 0.04 | 12.5 | 0.21 |
|  |  | Porifera sp. 02 | 89 | 0.2 | 31.25 | 0.96 |
|  |  | Porifera sp. 03 | 165 | 0.2 | 37.5 | 1.73 |
|  |  | Porifera sp. 04 | 1004 | 1.36 | 43.75 | 3.84 |
|  |  | Porifera sp. 05 | 122 | 0.24 | 28.75 | 1.07 |
|  |  | Porifera sp. 06 | 31 | 0.16 | 7.5 | 0.19 |
|  |  | Porifera sp. 11 | 26 | 0.08 | 10 | 0.21 |
|  |  | Porifera sp. 13 | 9 | 0.12 | 7.5 | 0.11 |
|  |  | Porifera sp. 15 | 56 | 0.2 | 20 | 0.75 |
|  |  | Porifera sp. 17 | 20 | 0.12 | 7.5 | 0.27 |
|  |  | Porifera sp. 19 | 803 | 0.8 | 57.5 | 4.03 |
|  |  | Porifera sp. 20 | 50 | 0.12 | 13.75 | 0.4 |
|  |  | Porifera sp. 24 | 30 | 0.24 | 11.25 | 0.35 |
|  |  | Porifera sp. 25 | 43 | 0.08 | 13.75 | 0.43 |
|  |  | Porifera sp. 27 | 24 | 0.2 | 8.75 | 0.11 |
|  |  | Porifera sp. 28 | 36 | 0.08 | 11.25 | 0.35 |
|  |  | Porifera sp. 29 | 12 | 0.04 | 2.5 | 0.03 |
|  |  | Porifera sp. 30 | 35 | 0.24 | 8.75 | 0.35 |
|  |  | Porifera sp. 40 | 1 | - | 1.25 | - |
|  |  | Porifera sp. 41 | 6 | 0.04 | 6.25 | 0.05 |
|  |  | Porifera sp. 45 | 110 | 0.12 | 40 | 1.6 |
|  |  | Porifera sp. 46 | 98 | 0.16 | 16.25 | 0.72 |
|  |  | Porifera sp. 47 | 92 | 0.32 | 32.5 | 1.07 |
|  |  | Porifera sp. 48 | 21 | 0.24 | 7.5 | 0.16 |
|  |  | Porifera sp. 49 | 1195 | 0.92 | 45 | 4.56 |
|  |  | Porifera sp. 54 | 7 | 0.08 | 2.5 | 0.03 |
|  |  | Porifera sp. 55 | 59 | 0.52 | 10 | 0.51 |
|  |  | Porifera sp. 57 | 306 | 2.56 | 18.75 | 0.99 |
|  |  | Porifera sp. 58 | 7 | 0.08 | 3.75 | 0.13 |
|  |  | Porifera sp. 59 | 72 | 0.52 | 7.5 | 0.4 |
|  |  | Porifera sp. 61 | 4 | 0.08 | 3.75 | 0.05 |
|  |  | Porifera sp. 62 | 2 | 0.04 | 2.5 | 0.03 |
|  |  | Porifera sp. 63 | 15 | 0.12 | 3.75 | 0.08 |
|  |  | Porifera sp. 64 | 2 | 0.04 | 1.25 | 0.03 |
|  |  | Porifera sp. 65 | 2 | 0.04 | 1.25 | 0.03 |
|  |  | Porifera sp. 66 | 2 | 0.04 | 2.5 | 0.05 |
|  |  | Porifera sp. 68 | 1 | 0.04 | 1.25 | 0.03 |
|  |  | Porifera sp. 69 | 1 | 0.04 | 1.25 | 0.03 |
|  |  | Porifera sp. 70 | 17 | 0.44 | 1.25 | 0.08 |
|  |  | Porifera sp. 71 | 21 | 0.28 | 1.25 | 0.16 |
|  | Homoscleromorpha | *Oscarella* spp. | 655 | 1.52 | 37.5 | 3.63 |
| Cnidaria | Anthozoa | *Adamsia palliata* | 1 | 0.04 | 1.25 | 0.03 |
|  |  | *Alcyonium acaule* | 242 | 1.28 | 32.5 | 1.71 |
|  |  | *Alcyonium coralloides* | 1617 | 1.68 | 47.5 | 6.54 |
|  |  | *Alcyonium palmatum* | 1205 | 0.64 | 46.25 | 10.76 |
|  |  | *Alicia mirabilis* | 2 | 0.04 | 2.5 | 0.03 |
|  |  | *Amphianthus* sp. | 1 | 0.04 | 1.25 | 0.03 |
|  |  | *Anemonia viridis* | 2 | 0.04 | 1.25 | 0.03 |
|  |  | *Antipathella subpinnata* | 303 | 0.88 | 12.5 | 1.07 |
|  |  | *Arachnanthus* sp. | 37 | 1.16 | 1.25 | 0.11 |
|  |  | *Caryophyllia* spp. | 254 | 0.28 | 38.75 | 1.71 |
|  |  | *Cereus pedunculatus* | 1 | 0.04 | 1.25 | 0.03 |
|  |  | *Cerianthus membranaceus* | 173 | 0.16 | 65 | 2.48 |
|  |  | *Cladocora caespitosa* | 1 | 0.04 | 1.25 | 0.03 |
|  |  | *Corallium rubrum* | 3302 | 10.16 | 20 | 2.05 |
|  |  | *Corynactis viridis* | 1869 | 12.12 | 2.5 | 0.19 |
|  |  | *Dendrophyllia cornigera* | 1449 | 2.96 | 13.75 | 2.7 |
|  |  | *Eunicella cavolini* | 12398 | 4.48 | 38.75 | 10.01 |
|  |  | *Eunicella singularis* | 307 | 0.68 | 15 | 2.38 |
|  |  | *Eunicella verrucosa* | 7010 | 3.08 | 75 | 24.21 |
|  |  | *Funiculina quadrangularis* | 12 | 0.04 | 7.5 | 0.19 |
|  |  | *Hoplangia durotrix* | 75 | 2.6 | 2.5 | 0.03 |
|  |  | *Kophobelemnon stelliferum* | 1 | - | 1.25 | - |
|  |  | *Leiopathes glaberrima* | 8 | 0.08 | 1.25 | 0.08 |
|  |  | *Leptogorgia sarmentosa* | 598 | 1.2 | 47.5 | 4.06 |
|  |  | *Leptopsammia pruvoti* | 30599 | 28.6 | 22.5 | 3.07 |
|  |  | *Paracyathus pulchellus* | 9 | 0.36 | 1.25 | 0.03 |
|  |  | *Paralcyonium spinulosum* | 5042 | 15.32 | 7.5 | 1.17 |
|  |  | *Paramuricea* cf. *macrospina* | 209 | 1.72 | 2.5 | 0.67 |
|  |  | *Paramuricea clavata* | 12186 | 3.76 | 66.25 | 15.35 |
|  |  | *Parantipathes larix* | 21 | 0.08 | 2.5 | 0.08 |
|  |  | *Parazoanthus axinellae* | 19318 | 4.08 | 76.25 | 22.36 |
|  |  | *Pennatula* sp. | 152 | 0.4 | 31.25 | 2.24 |
|  |  | *Phyllangia americana mouchezii* | 9 | 0.12 | 2.5 | 0.08 |
|  |  | *Polycyathus muellerae* | 3 | 0.04 | 3.75 | 0.05 |
|  |  | *Pteroeides spinosum* | 17 | 0.12 | 6.25 | 0.29 |
|  |  | *Savalia savaglia* | 26 | 0.64 | 2.5 | 0.03 |
|  |  | Sagartiidae | 6 | 0.08 | 3.75 | 0.08 |
|  |  | Scleractinia sp. 1 | 393 | 0.52 | 37.5 | 1.84 |
|  |  | Scleractinia sp. 2 | 390 | 1.36 | 33.75 | 2.35 |
|  |  | Scleractinia sp. 3 | 18 | 0.24 | 10 | 0.11 |
|  |  | Scleractinia sp. 4 | 160 | 1.76 | 6.25 | 0.27 |
|  |  | Scleractinia sp. 5 | 2 | 0.04 | 2.5 | 0.05 |
|  |  | Zoantharia | 10 | 0.4 | 1.25 | 0.03 |
|  |  | *Veretillum cynomorium* | 6 | 0.04 | 5 | 0.16 |
|  |  | *Virgularia mirabilis* | 5 | 0.12 | 2.5 | 0.08 |
|  | Hydrozoa | *Eudendrium* sp. | 84 | 0.28 | 6 | 0.32 |
|  |  | *Halecium halecium* | 1 | - | 1.25 | - |
|  |  | *Lytocarpia myriophyllum* | 2036 | 1.44 | 46.25 | 7.1 |
|  |  | *Nemertesia antennina* | 5 | 0.04 | 2.5 | 0.03 |
|  |  | *Sertularella grayi* | 78 | 0.64 | 2.5 | 0.32 |
|  |  | Hydrozoa sp. 1 | 816 | 0.72 | 72.5 | 5.82 |
|  |  | Hydrozoa sp. 2 | 547 | 1.48 | 23.75 | 1.79 |
|  |  | Hydrozoa sp. 3 | 17 | 0.16 | 6.25 | 0.08 |
|  |  | Hydrozoa sp. 4 | 11 | 0.16 | 3.75 | 0.08 |
|  |  | Hydrozoa sp. 5 | 66 | 0.12 | 7.5 | 0.24 |
| Platyhelminthes | Rhabditophora | *Prostheceraeus roseus* | 1 | 0.04 | 1.25 | 0.03 |
| Annelida | Echiuroidea | *Bonellia viridis* | 633 | 0.4 | 83.75 | 7.34 |
|  | Sabellida | *Acromegalomma* sp. | 52 | 0.12 | 22.5 | 0.67 |
|  |  | *Apomatus/Protula* complex | 260 | 0.24 | 48.75 | 3.02 |
|  |  | *Bispira viola* | 28502 | 110.32 | 21.25 | 1.12 |
|  |  | *Bispira* sp. | 3 | 0.08 | 2.5 | 0.05 |
|  |  | *Dialychone* sp. | 2722 | 39.04 | 3.75 | 0.29 |
|  |  | *Filograna/Salmacina* complex | 2599 | 3.28 | 78.75 | 12.57 |
|  |  | *Myxicola* sp. | 189 | 0.12 | 63.75 | 3.15 |
|  |  | *Sabella spallanzanii* | 116 | 0.12 | 47.5 | 1.47 |
|  |  | *Sabella* sp. | 205 | 2 | 3.75 | 0.05 |
|  |  | Sabellidae | 2363 | 38.28 | 38.75 | 2 |
|  |  | Serpulidae | 2511 | 5.28 | 80 | 6.35 |
|  | Terebellida | *Lanice conchilega* | 2 | 0.04 | 1.25 | 0.03 |
| Mollusca | Bivalvia | *Atrina* sp. | 12 | 0.24 | 3.75 | 0.16 |
|  |  | *Neopycnodonte cochlear* | 1478 | 9.8 | 12.5 | 0.67 |
|  |  | *Pecten* sp. | 10 | 0.12 | 6.25 | 0.21 |
|  |  | *Pinna nobilis* | 1 | 0.04 | 1.25 | 0.03 |
|  |  | *Pteria hirundo* | 9 | 0.04 | 8.75 | 0.19 |
|  |  | *Spondylus gaederopus* | 1 | 0.04 | 1.25 | 0.03 |
|  | Gastropoda | *Aporrhais pespelecani* | 1 | 0.04 | 1.25 | 0.03 |
|  |  | *Bolinus brandaris* | 2 | 0.04 | 2.5 | 0.05 |
|  |  | *Calliostoma zizyphinum* | 1 | 0.04 | 1.25 | 0.03 |
|  |  | *Euthria cornea* | 1 | 0.04 | 1.25 | 0.03 |
|  |  | *Facelina annulicornis* | 1 | 0.04 | 1.25 | 0.03 |
|  |  | *Felimare* sp. | 18 | 0.12 | 15 | 0.27 |
|  |  | *Flabellina* sp. | 15 | 0.12 | 6.25 | 0.13 |
|  |  | *Galeodea echinophora* | 6 | 0.24 | 2.5 | 0.03 |
|  |  | *Natica hebraea* | 1 | 0.04 | 1.25 | 0.03 |
|  |  | *Peltodoris atromaculata* | 9 | 0.08 | 5 | 0.08 |
|  |  | *Simnia spelta* | 1 | - | 1.25 | - |
|  |  | *Tritonia nilsodhneri* | 1 | - | 1.25 | - |
|  |  | Vermetidae | 12 | 0.32 | 5 | 0.13 |
| Arthropoda | Crustacea | *Anamathia rissoana* | 1 | - | 1.25 | - |
|  |  | *Calappa granulata* | 1 | 0.04 | 1.25 | 0.03 |
|  |  | *Corystes cassivelaunus* | 1 | 0.04 | 1.25 | 0.03 |
|  |  | *Ethusa mascarone* | 1 | 0.04 | 1.25 | 0.03 |
|  |  | *Galathea* sp. | 4 | 0.04 | 5 | 0.05 |
|  |  | *Homarus gammarus* | 4 | 0.04 | 3.75 | 0.03 |
|  |  | *Inachus* sp. | 12 | 0.04 | 12.5 | 0.27 |
|  |  | *Lysmata seticaudata* | 5 | - | 1.25 | - |
|  |  | *Maja squinado* | 2 | - | 2.5 | - |
|  |  | *Munida* sp. | 21 | 0.08 | 20 | 0.32 |
|  |  | *Mysis* sp. | 723 | 15.96 | 2.5 | 0.05 |
|  |  | Paguridae | 67 | 0.28 | 27.5 | 0.83 |
|  |  | *Palinurus elephas* | 108 | 0.24 | 32.5 | 1.04 |
|  |  | *Pilumnus hirtellus* | 1 | - | 1.25 | - |
|  |  | *Plesionika narval* | 512 | 0.56 | 6.25 | 0.03 |
|  |  | *Scyllarus arctus* | 1 | 0.04 | 1.25 | 0.03 |
|  |  | *Stenopus spinosus* | 1 | 0.04 | 1.25 | 0.03 |
| Phoronida |  | *Phoronis* sp. | 2 | 0.08 | 1.25 | 0.03 |
| Brachiopoda | Rhynchonellata | *Gryphus vitreus* | 86 | 1.16 | 1.25 | 0.05 |
|  |  | *Megerlia truncata* | 1337 | 8 | 2.5 | 0.27 |
| Bryozoa | Gymnolaemata | *Myriapora truncata* | 2207 | 2.8 | 71.25 | 9.23 |
|  |  | *Pentapora fascialis* | 1193 | 0.72 | 72.5 | 11.48 |
|  |  | *Reteporella* spp. | 1 | 0.04 | 1.25 | 0.03 |
|  |  | *Schizoporella* sp. | 2038 | 2.2 | 71.25 | 9.77 |
|  |  | *Smittina cervicornis/Adeonella calveti* | 550 | 1.56 | 18.75 | 1.07 |
|  |  | *Turbicellepora* sp. | 1169 | 0.76 | 65 | 8.81 |
|  | Stenolaemata | *Frondipora verrucosa* | 165 | 0.28 | 51.25 | 2.62 |
|  |  | *Hornera frondiculata* | 5 | 0.04 | 3.75 | 0.11 |
| Echinodermata | Asteroidea | *Astropecten aranciacus* | 1 | 0.04 | 1.25 | 0.03 |
|  |  | *Astropecten* sp. | 4 | 0.04 | 2.5 | 0.11 |
|  |  | *Chaetaster longipes* | 20 | 0.04 | 15 | 0.27 |
|  |  | *Coscinasterias tenuispina* | 3 | 0.04 | 3.75 | 0.05 |
|  |  | *Echinaster sepositus/Hacelia attenuata* | 522 | 0.2 | 73.75 | 7.47 |
|  |  | *Marthasterias glacialis* | 7 | 0.04 | 8.75 | 0.16 |
|  |  | *Ophidiaster ophidianus* | 2 | - | 2.5 | - |
|  |  | *Peltaster placenta* | 49 | 0.08 | 16.25 | 0.59 |
|  | Crinoidea | *Antedon mediterranea* | 408 | 1.64 | 6.25 | 0.99 |
|  |  | *Leptometra phalangium* | 27 | 0.12 | 8.75 | 0.45 |
|  | Echinoidea | *Centrostephanus longispinus* | 228 | 0.56 | 40 | 2.19 |
|  |  | *Cidaris cidaris/Stylocidaris affinis* | 2044 | 3.96 | 46.25 | 8.17 |
|  |  | *Echinus melo/Gracilechinus acutus* | 156 | 0.12 | 28.75 | 2.46 |
|  |  | *Spatangus purpureus* | 1 | 0.04 | 1.25 | 0.03 |
|  |  | *Sphaerechinus granularis* | 3 | 0.04 | 2.5 | 0.03 |
|  | Holothuroidea | *Holothuria* sp. | 2438 | 0.92 | 65 | 12.12 |
|  |  | *Parastichopus regalis* | 141 | 0.24 | 23.75 | 2.27 |
|  | Ophiuroidea | *Amphiura* sp. | 613 | 1.56 | 27.5 | 1.84 |
|  |  | *Astrospartus mediterraneus* | 263 | 0.32 | 30 | 2.46 |
|  |  | *Ophiothrix fragilis* | 1 | 0.04 | 1.25 | 0.03 |
|  |  | *Ophiura ophiura* | 1 | 0.04 | 1.25 | 0.03 |
| Chordata | Aplousobranchia | *Aplidium* sp. | 3 | 0.08 | 2.5 | 0.05 |
|  |  | *Clavelina* sp. | 23 | 0.28 | 5 | 0.11 |
|  |  | *Diazona violacea* | 1 | 0.04 | 1.25 | 0.03 |
|  |  | *Rhopalaea neapolitana* | 1 | 0.04 | 1.25 | 0.03 |
|  | Phlebobranchia | *Ciona* sp. | 37 | 0.32 | 22.5 | 0.48 |
|  |  | *Phallusia fumigata* | 10 | 0.12 | 6.25 | 0.13 |
|  |  | *Phallusia mammillata* | 1 | 0.04 | 1.25 | 0.03 |
|  | Stolidobranchia | *Halocynthia papillosa* | 1850 | 0.92 | 72.5 | 10.27 |
|  |  | *Microcosmus* sp. | 6 | 0.08 | 3.75 | 0.08 |

SU: sampling unit.
