# Supplementary material for: Megabenthic communities of the Ligurian deep continental shelf and shelf break (NW Mediterranean Sea)
Source: PLoS One. 2019 Oct 17;14(10):e0223949. doi: 10.1371/journal.pone.0223949 (PMC6797210; doi:10.1371/journal.pone.0223949)
Supplement: S4 Table — (DOCX) [file pone.0223949.s004.docx]

**S4 Table.** **Biological features of the 80 ROV dives carried out on the Ligurian continental shelf and shelf break.**

| **Dive** | **Nº of species** | **Av. nº spp. per SU ± SD** | **Max nº spp. in SU** | **Av. nº org. per SU ± SD** | **Max nº org. in SU** | **Av. expH per SU ± SD** | **Max. expH per SU** | **Dominant assemblage** | **Characteristic species** |
| --- | --- | --- | --- | --- | --- | --- | --- | --- | --- |
| A01 | 33 | 1.96 ± 1.90 | 8 | 3.3 ± 4.2 | 20 | 1.96 ± 1.37 | 6.92 | 1 | *Alcyonium palmatum* |
| A02 | 9 | 0.67 ± 0.82 | 3 | 1.4 ± 2.3 | 8 | 1.15 ± 0.39 | 2.65 | N/A | *C. cidaris/S. affinis* |
| A03 | 70 | 4.49 ± 3.45 | 14 | 18.9 ± 21.2 | 95 | 3.28 ± 2.15 | 8.71 | 6 | *Axinella* spp. |
| A04 | 80 | 6.93 ± 4.34 | 17 | 33.7 ± 28.9 | 129 | 4.37 ± 2.51 | 11.09 | 6 | *Paramuricea clavata* |
| A05 | 17 | 1.00 ± 1.31 | 3 | 1.0 ± 1.3 | 3 | 1.47 ± 0.87 | 3.00 | N/A | *Sarcotragus foetidus* |
| A06 | 60 | 3.64 ± 3.10 | 12 | 14.5 ± 14.6 | 60 | 2.55 ± 1.58 | 6.60 | 8 | *Eunicella verrucosa* |
| A07 | 61 | 2.41 ± 3.67 | 12 | 14.2 ± 26.6 | 140 | 2.15 ± 1.79 | 6.46 | 5 | *Axinella* spp. |
| A08 | 36 | 8.73 ± 3.23 | 14 | 92.9 ± 100.3 | 356 | 4.63 ± 1.83 | 7.68 | 4 | *Megerlia truncata* |
| B01 | 44 | 4.4 ± 2.84 | 11 | 49.9 ± 87.7 | 443 | 2.70 ± 1.68 | 8.24 | 4 | *Dialychone* sp. |
| B02 | 58 | 6.68 ± 3.11 | 13 | 74.0 ± 146.7 | 1048 | 3.40 ± 1.88 | 8.53 | 11 | *Haliclona* cf. *mediterranea* |
| B03 | 66 | 6.26 ± 5.45 | 21 | 42.3 ± 66.3 | 319 | 3.59 ± 2.07 | 8.12 | 11 | *Axinella* spp. |
| B04 | 51 | 9.29 ± 3.24 | 16 | 35.8 ± 20.3 | 84 | 6.05 ± 2.08 | 10.23 | 4 | *Axinella* spp. |
| B05 | 63 | 7.33 ± 5.35 | 15 | 45.0 ± 41.8 | 134 | 3.97 ± 2.28 | 7.05 | 3 | *Axinella* spp. |
| B06 | 32 | 2.14 ± 2.51 | 8 | 6.9 ± 11.8 | 45 | 2.09 ± 1.33 | 5.00 | 3 | *Axinella* spp. |
| B07 | 73 | 8.53 ± 5.10 | 18 | 45.7 ± 33.0 | 130 | 5.56 ± 3.22 | 12.86 | 3 | *Axinella* spp. |
| B08 | 21 | 2.90 ± 1.55 | 6 | 4.8 ± 2.3 | 11 | 2.67 ± 1.43 | 5.33 | 1 | *Lytocarpia myriophyllum* |
| C01 | 42 | 7.71 ± 3. 01 | 16 | 58.1 ± 56.5 | 347 | 4.58 ± 2.00 | 9.80 | 5 | *Eunicella cavolini* |
| C02 | 29 | 3.65 ± 1.63 | 6 | 14.9 ± 11.8 | 50 | 2.81 ± 1.23 | 5.12 | 9 | *Dendrophyllia cornigera* |
| C03 | 15 | 1.10 ± 1.11 | 6 | 3.31 ± 8.4 | 55 | 1.23 ± 0.63 | 4.71 | 1 | *Alcyonium palmatum* |
| C04 | 20 | 1.14 ± 1.88 | 7 | 8.20 ± 17.8 | 91 | 1.43 ± 0.83 | 3.64 | 3 | *Parazoanthus axinellae* |
| C05 | 22 | 3.41 ± 3.83 | 11 | 17.3 ± 23.3 | 87 | 2.80 ± 2.30 | 7.39 | 3 | *Eunicella verrucosa* |
| C06 | 9 | 1.03 ± 0.96 | 4 | 2.4 ± 3.1 | 12 | 1.29 ± 0.61 | 3.79 | 1 | *Pentapora fascialis* |
| D01 | 32 | 5.92 ± 3.42 | 14 | 33.6 ± 25.0 | 123 | 4.04 ± 1.98 | 8.48 | 6 | *Eunicella verrucosa* |
| D02 | 14 | 1.82 ± 1.04 | 5 | 3.9 ± 3.1 | 20 | 1.72 ± 0.83 | 4.75 | 1 | *Alcyonium palmatum* |
| D03 | 31 | 2.18 ± 2.23 | 11 | 13.1 ± 19.3 | 101 | 1.67 ± 1.05 | 6.14 | 6 | *Axinella* spp. |
| D04 | 8 | 1.49 ± 0.76 | 3 | 4.3 ± 2.6 | 13 | 1.41 ± 0.56 | 3.00 | 1 | *Alcyonium palmatum* |
| D05 | 37 | 5.95 ± 2.42 | 10 | 20.7 ± 14.5 | 64 | 4.32 ± 1.89 | 8.49 | 4 | *Eunicella cavolini* |
| D06 | 18 | 1.72 ± 1.97 | 7 | 6.6 ± 10.0 | 38 | 1.60 ± 0.95 | 4.07 | 4 | *Axinella* spp. |
| D07 | 24 | 2.53 ± 2.00 | 9 | 7.8 ± 8.2 | 32 | 2.25 ± 1.32 | 5.87 | 4 | *C. cidaris/S. affinis* |
| D08 | 53 | 2.62 ± 2.59 | 14 | 10.8 ± 17.9 | 92 | 2.08 ± 1.29 | 6.65 | 1 | *Alcyonium palmatum* |
| E01 | 70 | 9.62 ± 6.96 | 21 | 239.5 ± 251.4 | 794 | 2.78 ± 1.57 | 6.76 | 7 | *Leptopsammia pruvoti* |
| E02 | 70 | 18.63 ± 5.22 | 27 | 452. ± 224. | 979 | 5.85 ± 1.73 | 10.23 | 7 | *Leptopsammia pruvoti* |
| E03 | 45 | 4.97 ± 5.03 | 17 | 46.7 ± 57.6 | 185 | 2.62 ± 1.87 | 6.92 | 5 | *Axinella* spp. |
| E04 | 44 | 2.25 ± 3.80 | 12 | 32.5 ± 103. | 578 | 1.83 ± 1.59 | 7.57 | 3 | *Parazoanthus axinellae* |
| E05 | 38 | 3.77 ± 4.14 | 12 | 275. ± 748. | 2758 | 2.33 ± 1.65 | 5.97 | 6 | *Bispira viola* |
| E06 | 58 | 4.83 ± 4.44 | 14 | 174.5 ± 472.2 | 2327 | 2.81 ± 2.18 | 8.27 | 3 | *Bispira viola* |
| E07 | 49 | 4.91 ± 4.20 | 15 | 36.9 ± 44.9 | 153 | 2.82 ± 1.94 | 7.20 | 5 | *Eunicella cavolini* |
| E08 | 42 | 8.62 ± 3.28 | 14 | 81.7 ± 41.6 | 158 | 3.94 ± 1.72 | 7.35 | 5 | *Eunicella cavolini* |
| E09 | 30 | 3.29 ± 2.51 | 10 | 34.9 ± 22.0 | 83 | 1.92 ± 1.00 | 4.15 | 9 | *Dendrophyllia cornigera* |
| E10 | 52 | 5.05 ± 3.59 | 15 | 63.6 ± 77.4 | 391 | 2.97 ± 1.80 | 6.75 | 5 | *Paralcyonium spinulosum* |
| E11 | 65 | 5.92 ± 3.30 | 14 | 50.3 ± 37.8 | 193 | 3.20 ± 1.88 | 8.91 | 10 | *Paralcyonium spinulosum* |
| E12 | 63 | 6.46 ± 4.10 | 18 | 42.3 ± 40.3 | 155 | 4.17 ± 2.40 | 10.38 | 1 | *Paramuricea clavata* |
| E13 | 52 | 6.62 ± 4.19 | 15 | 26.1 ± 22.9 | 98 | 4.91 ± 2.97 | 10.65 | 1 | *Axinella* spp. |
| E14 | 52 | 8.24 ± 3.72 | 16 | 36.5 ± 26.5 | 121 | 5.46 ± 2.39 | 11.12 | 8 | *Eunicella verrucosa* |
| F01 | 71 | 6.24 ± 5.48 | 15 | 32.6 ± 39.8 | 146 | 4.00 ± 2.67 | 10.66 | 3 | *Axinella* spp. |
| F02 | 37 | 6.00 ± 4.72 | 15 | 36.4 ± 37.5 | 141 | 3.74 ± 2.49 | 11.98 | 3 | *Axinella* spp. |
| F03 | 20 | 0.73 ± 1.37 | 7 | 32.5 ± 186.2 | 1324 | 1.19 ± 0.65 | 5.00 | 12 | *Bispira viola* |
| F04 | 30 | 2.23 ± 2.30 | 8 | 11.9 ± 15.4 | 70 | 1.83 ± 1.14 | 5.40 | 8 | *Eunicella verrucosa* |
| F05 | 43 | 3.46 ± 3.25 | 11 | 28.4 ± 41.3 | 197 | 2.50 ± 1.54 | 6.56 | 3 | *Axinella* spp. |
| F06 | 46 | 3.71 ± 2.74 | 16 | 294.4 ± 685.4 | 2595 | 2.28 ± 1.33 | 5.86 | 8 | *Bispira viola* |
| G01 | 28 | 2.50 ± 2.73 | 8 | 5.74 ± 9.5 | 41 | 2.39 ± 1.82 | 7.19 | 3 | *Axinella* spp. |
| G02 | 24 | 0.71 ± 1.17 | 5 | 4.2 ± 17.6 | 176 | 1.24 ± 0.56 | 3.79 | 7 | *Leptopsammia pruvoti* |
| G03 | 46 | 4.51 ± 3.47 | 11 | 30.7 ± 34.7 | 171 | 2.74 ± 1.36 | 5.72 | 3 | *Axinella* spp. |
| G04 | 23 | 1.13 ± 1.64 | 7 | 2.3 ± 4.7 | 20 | 1.52 ± 1.04 | 5.99 | 3, 4 | *Axinella* spp. |
| G05 | 50 | 5.05 ± 3.99 | 11 | 61.2 ± 74.8 | 291 | 2.85 ± 1.53 | 6.21 | 3 | *Leptopsammia pruvoti* |
| H01 | 26 | 2.44 ± 1.89 | 8 | 8.7 ± 11.8 | 50 | 2.09 ± 1.11 | 5.74 | 1 | *Myriapora truncata* |
| H02 | 36 | 9.75 ± 2.53 | 14 | 186.8 ± 136.7 | 471 | 4.27 ± 1.11 | 6.19 | 6 | *Leptopsammia pruvoti* |
| H03 | 46 | 5.19 ± 3.69 | 12 | 67.4 ± 95.5 | 496 | 2.92 ± 1.52 | 6.75 | 3 | *Axinella* spp. |
| H04 | 37 | 1.98 ± 2.51 | 9 | 21.8 ± 55.2 | 318 | 1.59 ± 0.86 | 4.02 | 7 | *Leptopsammia pruvoti* |
| H05 | 30 | 2.36 ± 2.77 | 8 | 50.8 ± 103.1 | 283 | 1.63 ± 1.12 | 4.41 | 5 | *Corynactis viridis* |
| H06 | 39 | 1.18 ± 1.88 | 7 | 2.4 ± 5.0 | 28 | 1.56 ± 1.22 | 6.73 | 4 | *Axinella* spp. |
| H07 | 33 | 3.25 ± 3.03 | 8 | 10.1 ± 10.9 | 37 | 2.56 ± 1.78 | 6.92 | 4 | *Axinella* spp. |
| H08 | 46 | 2.71 ± 3.06 | 12 | 13.6 ± 18.1 | 66 | 2.11 ± 1.63 | 7.79 | 2 | *Lytocarpia myriophyllum* |
| H09 | 22 | 1.50 ± 1.66 | 7 | 2.2 ± 2.8 | 12 | 1.59 ± 1.23 | 5.86 | 1 | *Sabellidae* |
| H10 | 69 | 8.12 ± 5.70 | 19 | 78.3 ± 77.5 | 237 | 4.10 ± 2.86 | 12.43 | 5 | *Axinella* spp. |
| H11 | 53 | 5.19 ± 3.85 | 19 | 36.1 ± 42.7 | 175 | 3.04 ± 1.67 | 8.07 | 2 | *Eunicella cavolini* |
| H12 | 52 | 3.45 ± 3.26 | 14 | 34.9 ± 128.5 | 871 | 2.54 ± 1.52 | 6.61 | 3 | *Leptopsammia pruvoti* |
| H13 | 51 | 7.31 ± 4.47 | 14 | 58.6 ± 65.3 | 260 | 4.06 ± 2.08 | 8.26 | 5 | *Axinella* spp. |
| I01 | 42 | 4.38 ± 3.24 | 12 | 33.6 ± 46.5 | 193 | 3.02 ± 1.70 | 7.31 | 3 | *Parazoanthus axinellae* |
| I02 | 31 | 2.49 ± 2.66 | 9 | 8.5 ± 13.7 | 60 | 2.31 ± 1.58 | 6.13 | 6 | *Paramuricea clavata* |
| I03 | 39 | 3.40 ± 3.43 | 14 | 9.9 ± 11.3 | 40 | 2.90 ± 2.31 | 11.07 | 6 | *Paramuricea clavata* |
| I04 | 40 | 2.97 ± 3.50 | 11 | 10.8 ± 16.5 | 54 | 2.59 ± 2.23 | 8.44 | 8 | *Axinella* spp. |
| I05 | 9 | 0.40 ± 0.92 | 5 | 0.6 ± 1.3 | 6 | 1.14 ± 0.63 | 5.00 | N/A | *Alcyonium palmatum* |
| I06 | 30 | 2.09 ± 3.04 | 11 | 22.1 ± 57.2 | 252 | 1.85 ± 1.88 | 8.45 | 1 | *Neopycnodonte cochlear* |
| I07 | 43 | 4.72 ± 4.16 | 13 | 17.5 ± 19.1 | 89 | 3.65 ± 2.48 | 8.83 | 3 | *Axinella* spp. |
| I08 | 26 | 2.76 ± 2.37 | 9 | 8.0 ± 9.0 | 31 | 2.50 ± 1.56 | 6.75 | 3 | *Parazoanthus axinellae* |
| J01 | 46 | 6.76 ± 3.07 | 14 | 90.2 ± 93.6 | 419 | 3.60 ± 1.45 | 7.03 | 3 | *Axinella* spp. |
| J02 | 43 | 6.71 ± 3.91 | 12 | 37.3 ± 35.6 | 109 | 4.12 ± 2.01 | 8.21 | 8 | *Eunicella verrucosa* |
| J03 | 11 | 0.33 ± 1.00 | 4 | 0.5 ± 1.5 | 7 | 1.19 ± 0.61 | 3.79 | N/A | *Pecten* sp. |
| J04 | 31 | 5.94 ± 2.21 | 8 | 34.6 ± 25.6 | 90 | 3.98 ± 1.23 | 5.83 | 3 | *Parazoanthus axinellae* |

Areas A-J can be identified in Fig 1. SU: sampling unit.
